# Patient-specific hepatocyte-like cells derived from induced pluripotent stem cells model pazopanib-mediated hepatotoxicity

**DOI:** 10.1038/srep41238

**Published:** 2017-01-25

**Authors:** Yukti Choudhury, Yi Chin Toh, Jiangwa Xing, Yinghua Qu, Jonathan Poh, Li Huan, Hui Shan Tan, Ravindran Kanesvaran, Hanry Yu, Min-Han Tan

**Affiliations:** 1 Institute of Bioengineering and Nanotechnology, 31 Biopolis Way, Nanos #04-01, Singapore 138669, Republic of Singapore; 2 Department of Biomedical Engineering, Faculty of Engineering, National University of Singapore, 4 Engineering Drive 3, E4 #04-08, Singapore 117583, Republic of Singapore; 3 Division of Medical Oncology, National Cancer Centre, Singapore 169610, Republic of Singapore; 4 Yong Loo Lin School of Medicine and Mechanobiology Institute, National University of Singapore, Republic of Singapore; 5 Gastroenterology Department, Nanfang Hospital, Southern Medical University, Guangzhou 510515, China

## Abstract

Idiosyncratic drug-induced hepatotoxicity is a major cause of liver damage and drug pipeline failure, and is difficult to study as patient-specific features are not readily incorporated in traditional hepatotoxicity testing approaches using population pooled cell sources. Here we demonstrate the use of patient-specific hepatocyte-like cells (HLCs) derived from induced pluripotent stem cells for modeling idiosyncratic hepatotoxicity to pazopanib (PZ), a tyrosine kinase inhibitor drug associated with significant hepatotoxicity of unknown mechanistic basis. *In vitro* cytotoxicity assays confirmed that HLCs from patients with clinically identified hepatotoxicity were more sensitive to PZ-induced toxicity than other individuals, while a prototype hepatotoxin acetaminophen was similarly toxic to all HLCs studied. Transcriptional analyses showed that PZ induces oxidative stress (OS) in HLCs in general, but in HLCs from susceptible individuals, PZ causes relative disruption of iron metabolism and higher burden of OS. Our study establishes the first patient-specific HLC-based platform for idiosyncratic hepatotoxicity testing, incorporating multiple potential causative factors and permitting the correlation of transcriptomic and cellular responses to clinical phenotypes. Establishment of patient-specific HLCs with clinical phenotypes representing population variations will be valuable for pharmaceutical drug testing.

Idiosyncratic drug-induced hepatotoxicity is a major real-world clinical concern that leads to considerable morbidity, mortality, treatment dose attenuation and interruption, and is also a major reason behind withdrawal of drugs from market^1^. Due to these unpredictable adverse drug-related events, promising drugs and drug candidates are likely to fail to reach full efficacy in the patient population. The identification of individuals at risk for such hepatotoxicity, together with a context-specific understanding of the mechanism of hepatotoxicity would be of considerable clinical and industry interest. To date, no suitable models for idiosyncratic hepatotoxicity have been developed, the difficulties compounded by rarity and inevitable retrospective nature of investigation in the clinical setting. The current gold standard *in vitro* model for traditional hepatotoxicity testing is primary human hepatocytes (PHP)^2^,^3^, but being representative of specific individual genetic backgrounds they are unsuitable for modeling inter-individual variability in drug response in a clinically relevant context. Conventional models ignore patient features, including genetic polymorphisms that are major susceptibility factors to a drug, whose metabolic and physicochemical properties primarily determine the basis for such toxicity^4^.

In an effort to incorporate patient-specific features in the study of idiosyncratic hepatotoxicity, the use of induced pluripotent stem cells (iPSCs) which retain host features including predisposing genetic risk factors has been proposed^5^,^6^. The ability to differentiate iPSCs to hepatocyte-like cells (HLCs) with appropriate drug metabolizing capacities^7^,^8^,^9^, has made general toxicity assessments on these liver-derived cells feasible^10^,^11^,^12^,^13^,^14^. HLCs have been extensively characterized^15^ which has led to their improved functionality as drug-metabolizing cells^16^,^17^. Still, the demonstration of iPSC-derived HLCs as models for idiosyncratic hepatotoxicity has thus far lacked clear clinical relevance and has been limited to metabolic variations attributable to single well-characterized cytochrome P450 variants^18^ or to the observation of donor-dependent differences in drug toxicity screens^19^. In a recent study, toxicity to valproic acid was modeled in iPSC-derived HLCs from two individuals with Alpers sydrome, characterized by mutations in *POLG* and increased sensitivity to valproic acid^20^. In another study, HLCs derived from alpha-1 antitrypsin (AAT)-deficient patient-specific iPSCs exhibited mutant AAT protein accumulation and autophagic flux reminiscent of the clinical disease^21^. These studies highlighted a known relationship between a specific rare genetic mutation and phenotype as reflected in patient-specific HLCs. However, outside such rare disorders, mechanisms of drug-induced hepatotoxicity are poorly understood in the full patient context^22^, especially as single common risk variants have been found to have limited association with drug-induced hepatotoxicity^23^. iPSCs and their cellular derivatives are genetic matches to their donors, and for donors with clinically identified hepatotoxicity provide unprecedented opportunity for retrospective mechanistic investigation that comprehensively encompasses the multitude of potential risk factors.

As new classes of drugs are introduced, the inevitability of idiosyncratic adverse drug reactions becomes apparent^24^. Therefore, towards fulfilling an unmet clinical need and harnessing the potential utility of patient-specific HLCs, we demonstrate as proof-of-concept the modeling of idiosyncratic hepatotoxicity to drugs with uncharacterized basis and mechanisms of toxicity. In this study, we have attempted a retrospective reconstruction of adverse hepatic reactions to pazopanib (PZ, Votrient^®^, GlaxoSmithKine), a clinically efficacious drug commonly prescribed for the treatment of advanced renal cell carcinoma^25^,^26^, but with a high incidence of hepatotoxicity^27^,^28^. Grade 3–4 elevation of serum alanine aminotransferase (ALT) and/or aspartate aminotransferase (AST) in 12–17%, and isolated hyperbilirubinemia in 36% of patients is observed at administered dose of 800 mg daily. PZ belongs to the class of tyrosine kinase inhibitor drugs, several among which are known to be metabolized to reactive intermediates partially accounting for their toxicity profiles^29^. However, for PZ no reactive metabolites have been identified in patients^30^, and pharmacogenetic analyses have yielded weak associations with two genetic markers *HFE*^31^ and *UGT1A1*^32^ for elevated ALT and bilirubin levels, respectively. The high incidence of hepatotoxicity, the obscure mechanism underlying this and the paucity of biomarkers for predicting hepatotoxicity, prompted the choice of PZ as the drug to demonstrate the utility of patient-specific HLCs for modeling idiosyncratic hepatotoxicity.

We generated iPSCs from blood-isolated lymphocytes of patients who suffered hepatotoxic side effects from PZ treatment clinically, and differentiated them into functional HLCs (HT-HLCs). Equivalent HLCs were derived from patients who also received PZ but did not have any hepatotoxic side effects to serve as real-world controls (NHT-HLCs). Comparison of *in vitro* effects of PZ on the two groups of HLCs confirmed greater sensitivity of HT-HLCs to PZ toxicity compared to NHT-HLCs. Affirming the drug-specific nature of this observation, similar high-level toxicity was seen for all HLCs with the paradigm hepatotoxic drug, acetaminophen. Given the ability to infinitely expand HLCs from patient-specific iPSCs, we went on to perform transcriptomics analysis and show that oxidative stress is a potential mechanism by which PZ induces damage in these HLCs. Further comparative transcriptional analysis provided evidence of differential iron metabolism and more extensive oxidative damage in susceptible HLCs to account for the inter-individual variability in PZ-induced hepatotoxicity.

This is the first demonstration that patient-matched HLCs, even in the absence of explicit knowledge of genetic variations can recapitulate cellular phenotypes corresponding to variation in the adverse effects for a drug. Our results strengthen the case for iPSC-derived HLCs as a platform for modeling idiosyncratic hepatotoxicity that allows the interrogation of toxicity mechanism in the appropriate background of patient-specific features, with multiple causative factors at play. Of considerable pharmaceutical interest, the establishment of patient-specific HLCs with known clinical phenotypes, as parts of cell banks, with varied genetic backgrounds and range of drug sensitivities, can also aid in population level drug testing.

## Results

### Generation and characterization of hepatocyte-like cells (HLCs) from patients with differential clinical hepatotoxicity to pazopanib (PZ)

Five patients that received standard dosing (800 mg daily) of PZ treatment for metastatic renal cell cancer (RCC) were selected and consented to this study (Supplementary Table 1). Liver function monitoring post initiation of treatment showed that two patients tolerated the treatment (NHT1, NHT2) while three patients suffered clinical hepatotoxicity (HT1, HT2, HT3) as observed from the on-treatment high-grade elevations of ALT and AST levels (≥3 × upper limit of normal) (Supplementary Fig. 1). Sequencing of patient genomic DNA did not reveal any link with variants in *HFE*^31^ and *UGT1A1*^32^, reported to be associated with PZ-induced ALT and bilirubin elevation, respectively (Supplementary Table 2), suggesting that other undefined patient factors play a predisposing role in developing hepatotoxicity to PZ.

In order to more comprehensively capture patient features, iPSCs were generated for each patient from their EBV-immortalized B-lymphocytes (EBVi) by episomal reprogramming^33^,^34^ with *OCT4, SOX2, KLF4, SV40/LT, NANOG* and *LIN28* (Supplementary Fig. 2). Each patient-specific iPSC line (HT1, HT2, HT3, NHT1, NHT2), expressed OCT4, NANOG, TRA-1–60 and SSEA-4 which were not detectable in the EBVi lines they were derived from (Supplementary Fig. 3a and b). The ability to form three germ layers was confirmed for each iPSC line by *in vitro* differentiation to embryoid bodies and *in vivo* teratoma formation (Supplementary Fig. 3c and d). All iPSC lines showed normal karyotype, except HT1 iPSCs (Supplementary Fig. 3e), however, given equivalent growth rates, differentiation potentials (both embryoid bodies and below) of all iPSC lines, we did not consider this abnormality to be of consequence. Thus, iPSCs were successfully generated from cells isolated from all five patients that received PZ treatment.

**Figure 1:**
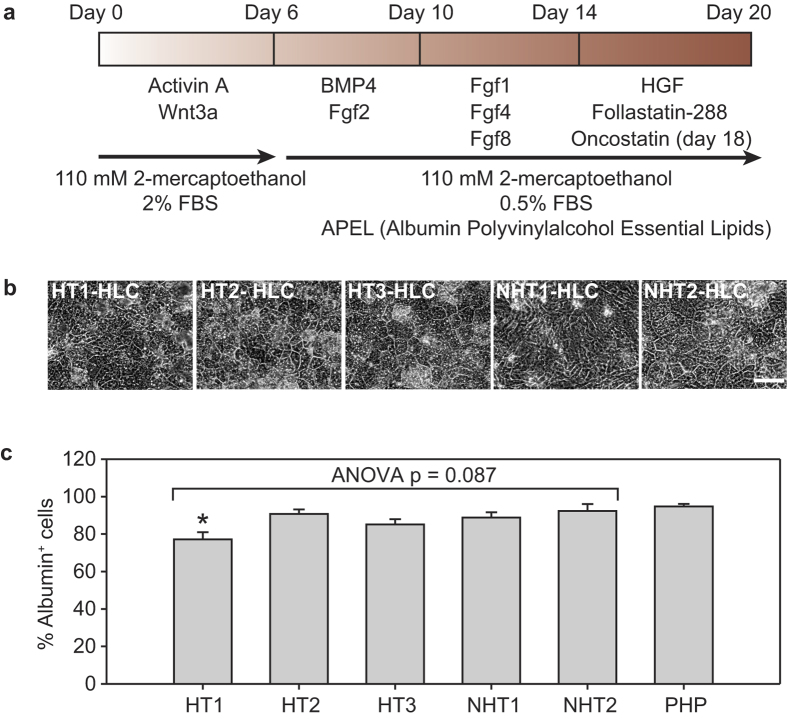
Differentiation of patient-specific iPSCs into hepatocyte-like cells (HLCs). (**a**) A four-step differentiation protocol spanning 20 days was used to derive HLCs from iPSCs. (**b**) Phase contrast images of iPSC-derived HLCs after 20 days of hepatic differentiation. Scale bar = 100 μm. (**c**) Percentage of albumin^+^ cells in different patient-specific iPSC-derived HLCs on day 20. Data are average ± s.e.m of at least 3 independent differentiation experiments initiated at distinct timepoints. *p < 0.05 compared to primary human hepatocytes (PHP) control (Bonferroni t-test).

We then generated hepatocyte-like cells (HLCs) from the patient iPSCs to serve as *in vitro* models to assess PZ-induced hepatotoxicity. A four-step differentiation protocol spanning 20 days was adopted from Roelandt *et al*. with modification^35^ (Fig. 1a). After 20 days of differentiation, majority of the cells adopted an epithelial cell morphology resembling hepatocytes (Fig. 1b) and exhibited a high proportion of albumin^+^ cells, ranging from 77.2% (HT1) to 92.3% (NHT2) (Fig. 1c and Supplementary Fig. 4a), comparable to the proportion of albumin^+^ cells reported for other iPSC-derived HLCs^7^. In undifferentiated iPSCs from both HT and NHT cases this proportion was very low (<1.5%) (Supplementary Fig. 4b). The percentage of albumin^+^ cells in iPSC-derived HLCs (referred to as “HLCs” hereafter) was not significantly lower than that of the PHP control except for HT1-HLCs (Bonferroni corrected t-test p = 0.008), but this measure was highly comparable among all the HLCs themselves, including across HT and NHT groups (ANOVA p = 0.087) (Fig. 1c).

**Figure 2:**
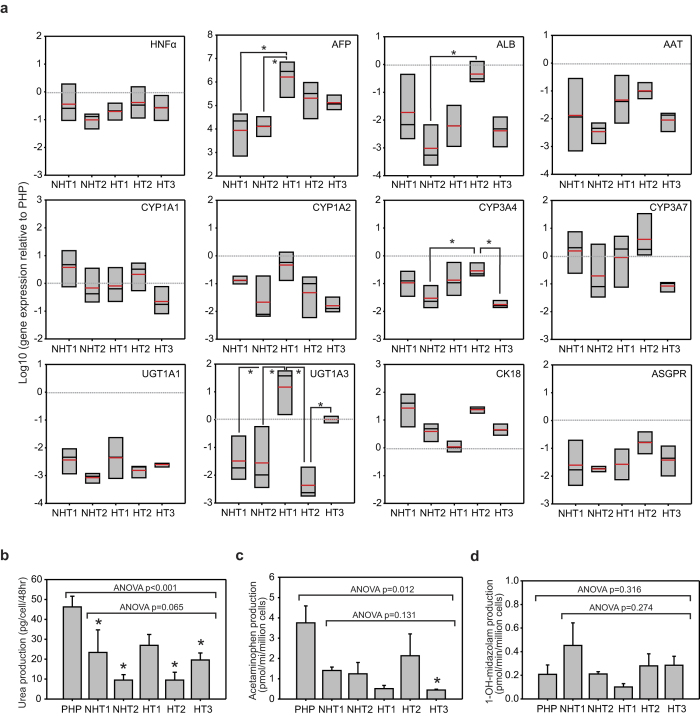
Characterization of liver-specific functions in patient-specific iPSC-derived HLCs. (**a**) Gene expression of hepatic markers. Box plots with mean values shown in red. n = at least 3 independent differentiation experiments. Expression is benchmarked to that in primary human hepatocytes (PHP) which is indicated by grey line. (**b**) Urea production. (**c**) Conversion of phenacetin by CYP1A2 enzymatic activity. (**d**) Conversion of midazolam by CYP3A4 enzymatic activity. Data in (**b–d**) are average ± s.e.m of at least 3 independent differentiation experiments initiated at distinct timepoints. *p < 0.05 compared to PHP control (Bonferroni t-test).

We next examined the expression of liver-specific markers in HLCs. When compared to freshly-thawed cryopreserved PHPs, the overall gene expression suggested that all HLCs exhibited a more fetal-like phenotype as indicated by their lower expressions of mature hepatocyte markers, such as *ALB, AAT, ASGPR, CYP1A2, CYP3A4, UGT1A1, UGT1A3* and higher expressions of hepatoblast or fetal hepatocyte markers, *HNF4α, AFP, CK18* and *CYP3A7*^36^ (Fig. 2a). This is consistent with previous reports that stem cell-derived HLCs have fetal hepatocyte-like phenotypes^37^. We observed that the gene expressions of liver-specific markers varied by up to 2 orders of magnitude between the 5 iPSC-HLC lines, which were similar to the extent of variations observed by Kajiwara *et al*., where they reported that intrinsic donor variability is a strong determinant of differentiation propensity of iPSCs to hepatocytes, measured as expression of liver-related genes^38^. Single factor ANOVA analysis indicated that there were no significant differences among the different HLC lines for all the genes tested, except for *AFP, ALB, CYP3A4* and *UGT1A3,* which differed significantly between disparate pairs of HLC lines (Bonferroni corrected t-test p < 0.05), without any specific distinction between HT and NHT lines. We conclude that apparent differences in hepatic differentiation capacity of distinct iPSC lines were largely masked by the large variations in gene expression levels within a single iPSC line, often by an order of magnitude (Fig. 2a).

We also measured the liver-specific functions of the HLCs. Urea production by all five HLCs was approximately 20–50% of PHP’s production rate (Fig. 2b), and were all significantly lower than PHP (p < 0.05, Bonferroni t-test) with the exception of HT1-HLCs. We measured the activity levels of two major cytochrome P450 (CYP) isoforms in the human liver, CYP1A2 and CYP3A4. The CYP1A2 activities of HLCs, ranging from 11–56%, were not significantly different from that of PHP except for HT3-HLCs (p < 0.05, Bonferroni t-test) (Fig. 2c). The CYP3A4 metabolic activities, ranging from 48–217%, were also similar to that of PHP (Fig. 2d), despite *CYP3A4* gene expression in HLC lines being between 2–32% of PHP (Fig. 2a). The disparity between expression level and metabolic activity of CYP3A4 could be due to its functional regulation at post-trascriptional level^39^,^40^. Notwithstanding these differences, we anticipate that HLCs from all five patient-specific iPSC lines will be capable of metabolizing PZ as CYP3A4 and CYP1A2 activities primarily mediate metabolism of PZ^30^.

Taken together, the results demonstrate that all patient specific-iPSC lines could differentiate into functional HLCs. There were inter-cell line and intra-cell line variability in liver specific marker expressions and functions. However, more variability was observed in gene expression than manifested in functional studies. Importantly, we could not distinguish between the HT and NHT lines based on the liver-specific marker expressions and functions. This buttresses our initial postulation that PZ-induced hepatotoxicity cannot easily be identified from single or a few variants in liver-specific markers or functions.

### Patient-specific HLCs model differential PZ-induced hepatotoxicity *in vitro*

**Figure 3:**
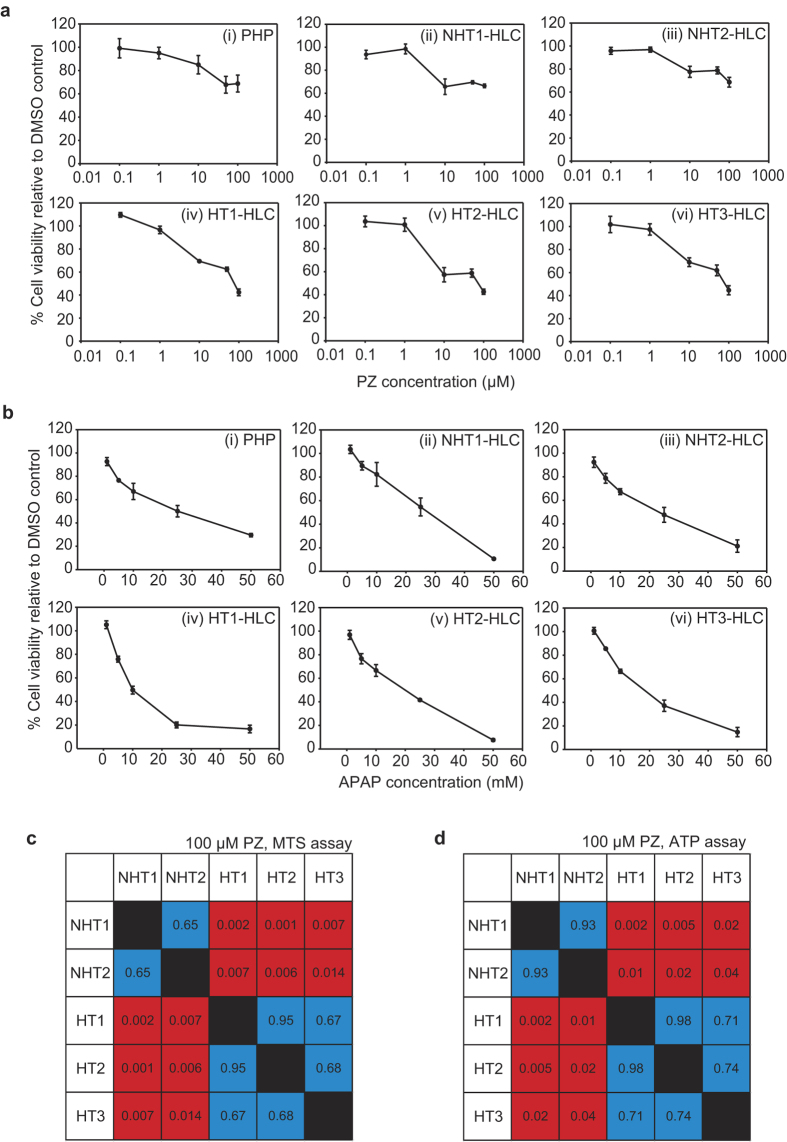
NHT and HT-iPSC derived HLCs exhibit differential *in vitro* hepatotoxicity to PZ but not to acetaminophen. (**a**) Cell viability of PHP(i) and HLCs (ii–vi) measured after 24 hours incubation with different concentrations of PZ. HLCs derived from iPSCs of patients without clinical PZ toxicity, NHT1 and NHT2 (ii,iii). HLCs derived from iPSCs from patients with clinical hepatotoxicity HT1, HT2 and HT3 (iv–vi). **(b)** Cell viability of PHP (i) and patient-specific HLCs (ii–vi) measured by MTS assay after 24 hours incubation with different concentrations of acetaminophen (APAP). In (**a** and **b**) data are means ± s.e.m. of 3 independent differentiation experiments. (**c**) Tabulation of pair-wise comparisons of cell viability measured by MTS assay of different patient-specific HLCs after treatment with 100 μM PZ. (**d**) Pair-wise comparisons of cell viability of HLCs measured by ATP assay. p-values of paired t-tests are indicated. Significantly different pairs based on Bonferroni corrected t-test (p < 0.05) are labeled red. Non-significantly different pairs are labeled blue.

We then assessed whether differential PZ-induced hepatotoxicity can be detected *in vitro* in patient-specific HLCs. HLCs were harvested after hepatic differentiation and plated onto 96-well plates and dosed with PZ at five different concentrations, ranging from 0.1–100 μM (Fig. 3a). This range of concentrations covered the reported cellular IC_50_ values for various cell lines and is close to the reported C_max_ value in human (*i.e.,* 122 μM)^41^ within the solubility limit of PZ in aqueous solution. We also tested the HLCs with a paradigm hepatotoxin, acetaminophen (APAP), to serve as a positive control drug (Fig. 3b). The cells were incubated for 24 hours with the drugs before hepatotoxicity effects were evaluated by measuring the cellular metabolic activity using MTS assay.

We observed that the hepatotoxic effects of PZ were less severe than APAP. For collagen-plated PHP controls, whereas APAP resulted in a clear dose-dependent toxicity (Fig. 3b(i)), cell viability was still 68.7 ± 7.3% even at the highest concentration tested for PZ (Fig. 3a(i)). HLCs derived from all five patient iPSC lines exhibited similar dose-dependent responses to APAP as PHP (Fig. 3b) but not to PZ (Fig. 3a). At low PZ concentrations (<1 μM), the HLCs did not show appreciable toxicity, with cell viability remaining at >95% without significant differences among HLCs (ANOVA, p = 0.95) (Fig. 3a). However, at 100 μM PZ, there was a significant difference in the cell viability of different HLC lines (ANOVA, p < 0.001). Cell viability for NHT1- and NHT2-HLCs were 66.4 ± 1.5% and 68.6 ± 4.3% respectively (Fig. 3a(ii,iii)). In comparison, HLCs derived from patients with clinical hepatotoxicity had lower cell viabilities of 42.3 ± 2.9% (HT1), 42.6 ± 2.3% (HT2), and 44.6 ± 3.2% (HT3) (Fig. 3a(iv–vi)). We performed post-hoc analysis using pair-wise t-test with Bonferroni’s correction to compare the different HLCs. There were no significant differences *within* the hepatotoxic and non-hepatotoxic groups but the differences in cell viability *between* the hepatotoxic and non-hepatotoxic groups were all statistically significant (Fig. 3c).

The differences in PZ-induced toxicity were observable only in HLCs and did not arise from intrinsic differences in the properties of the undifferentiated iPSC lines themselves, as no differential toxicity was observed between NHT and HT iPSC lines. All 5 undifferentiated iPSC lines were significantly more sensitive to both PZ (Supplementary Fig. 5) and APAP (Supplementary Fig. 6), compared to their differentiated HLC counterparts, and to similar extents compared to one another. To confirm that the differential response of the patient iPSC-derived HLCs to PZ-induced hepatotoxicity was not specific to the MTS assay, we performed drug testing with another assay that measures intracellular ATP levels to indicate cytotoxicity effects. We observed that there was once again a significant difference between the HLCs at 100 μM PZ (ANOVA, p = 0.002), where approximately 66% of NHT1- and NHT2-derived HLCs remained viable while only 45–47% of HT1-, HT2- and HT3-HLCs survived (Supplementary Fig. 7). Post-hoc analysis for ATP assay showed similar results as the MTS assay (Fig. 3d). These results indicated that the 20% differential cytotoxic response between the NHT- and HT-HLCs was likely a manifestation of the intrinsic difference in their susceptibility to PZ-induced hepatotoxicity, and not due to assay-dependent fluctuations or due to differences in the potentials of the iPSC lines. This demonstrated that the patient-specific HLCs could recapitulate clinical PZ-induced idiosyncratic hepatotoxicity in an *in vitro* assay.

### Transcriptional changes due to PZ exposure have elements of oxidative stress common to all HLCs

**Figure 4:**
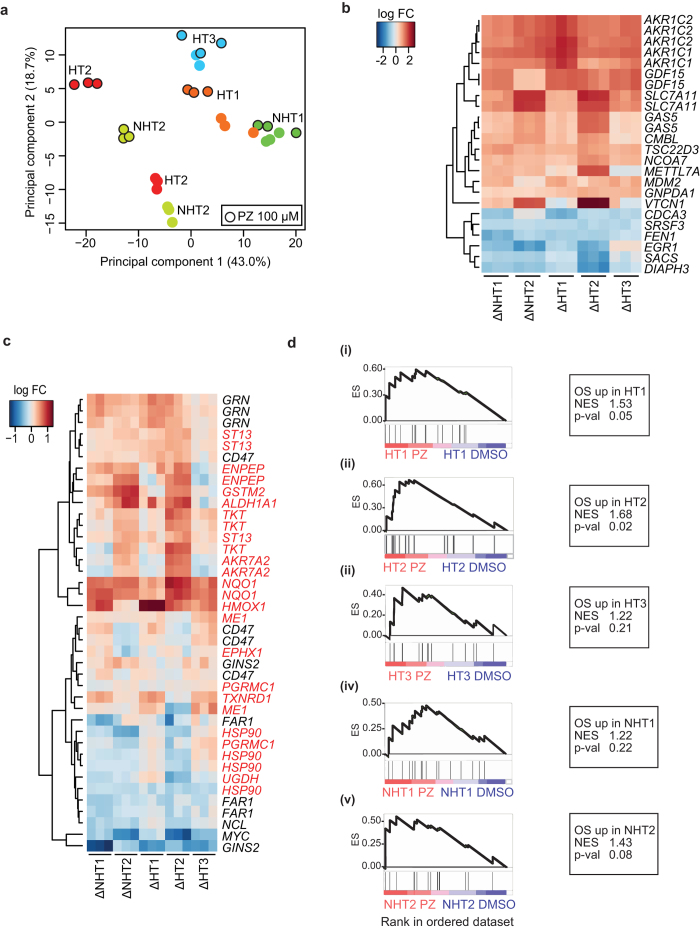
Transcriptional changes induced by PZ in HLCs have universally conserved elements related to oxidative stress. (**a**) Principal component analysis of expression data measured by Affymetrix microarray along the first principal component (PC1) and second principal component (PC2) axes. HLCs were treated with 100 μM PZ, represented by black outline, or with DMSO as control. Biological replicates of distinct origin and treatment type contribute to the dominant clusters. A shift in PC2 is seen for all HLCs with PZ treatment compared to their controls. (**b**) Heatmap visualizing expression levels of genes that undergo the largest PZ-induced changes in HLCs from HT and NHT groups. Top differentially expressed genes were identified for HT-HLCs and for NHT-HLCs and 24 probes corresponding to the union of these two gene lists are depicted. For each HLC, Δ indicates individual log2 fold-changes (log FC) relative to respective control. Each column corresponds to one of three biological replicates for each HLC line. (**c**) Heatmap of expression of genes in HLCs corresponding to a hepatocyte drug-induced oxidative stress (OS) signature derived from rat liver. Gene names in red are those for which an induction of expression is expected from original rat liver data. (**d**) GSEA enrichment plots for upregulated genes from OS signature (OS up) in HT-HLCs (i–iii) and NHT-HLCs (iv-v). For each HLC, all genes from expression dataset were rank-ordered according to their fold change between PZ- and control samples. Genes from the ‘OS up’ gene set are indicated by black bars and their rank in rank-ordered expression set suggests a strong bias for their upregulation in PZ-treated samples, reflected by the running enrichment score (ES) that peaks to the left of the plot. The normalized enrichment score (NES) and p-value of significance of enrichment are indicated for each plot.

Transcriptional changes that occur in hepatocytes can aid the understanding of the mechanistic basis for a drug’s hepatotoxic potential^42^. We availed the five HLC lines to investigate the mechanism behind PZ-induced hepatotoxicity, specifically the enhanced cytotoxic susceptibility of HT-HLCs to PZ. To achieve this we extracted RNA from each HLC line treated either with DMSO as control or with 100 μM PZ for 24 hours, and subjected the samples to microarray analysis. Dosing at this concentration was anticipated to produce a significant transcriptional signal in all HLCs given that cytotoxicity was observed in all HLCs, although it was distinctly lower in NHT-HLCs. Principal component analysis of expression data revealed that HLCs differ dramatically in their baseline transcriptional profiles, although treatment with PZ does generate a distinct shift in second principal component for all HLCs (Fig. 4a). To answer if gene expression changes, common and exclusive to HT1–3, could account for their greater susceptibility to PZ, first, the most significantly differentially expressed genes in each HLC line were identified (Supplementary Table 3), and analyzed for overlaps (Supplementary Fig. 8). This and subsequent grouped analysis for HT-HLCs and NHT-HLCs showed that irrespective of the HLCs’ PZ sensitivity, the largest gene expression changes due to PZ, including *SLC7A11, AKR1C1, AKR1C2* and *GDF15*, occurred in similar directionality and magnitude in all HLCs (Fig. 4b). Expression of selected genes from differential expression analysis was verified using qRT-PCR showing good agreement with the microarray platform (Supplementary Fig. 9). It was noted that many of the induced genes have documented functions in responding to cellular oxidative stress (OS), including *AKR1C1* and *AKR1C2*^43^ and *SLC7A11*^44^, that are known to be induced by Nrf2, a transcription factor central to cellular defense against oxidative stress^45^. Furthermore, *GDF15* and *EGR1* belong to a four-gene consensus signature of drug-induced hepatotoxicity developed from large-scale toxicogenomics data^46^.

In addition, we used the gene set enrichment analysis method (GSEA)^47^ to investigate coordinated functional changes induced in HLCs by PZ. All HLCs exhibited similar induction of IFN-α/IFN-γ response and bile acid metabolism, and repression of MYC and E2F targets and TGFβ signaling (Supplementary Table 4), reinforcing the notion of a universal effect of PZ in HLCs.

Toxicogenomic studies have shown that at least in the rat liver, oxidative stressors can be detected by the induction of a gene expression signature, distinguishable from other classes of hepatotoxicants and representative of Nrf2 activation^48^. As Nrf2-dependent response to OS is likely a conserved function in mammals, we translated a published signature corresponding to hepatotoxicant-induced OS in rat liver^49^ to homologous human genes and visualized changes to their expression in PZ-treated HLCs. Confirming the OS inducing effects of PZ on HLCs, the majority of the genes from the signature followed the expected pattern of induction or repression, and the overlap was much clearer for induced genes (Fig. 4c). Well-known targets of Nrf2 activity including *NQO1, HMOX1, AKR7A2* and *ALDH1A1* were clearly induced in most if not all HLCs. Among genes that did not have strong induction across all lines (including *HSP90* and *UGDH*), minor induction was still observed in at least two out of five HLCs. Some genes including *GRN* and *CD47,* that are repressed in rat liver upon OS showed discrepant induction in HLCs. We cannot rule out a non-specific or a rat liver-specific modulation of repressed genes. The expression of several Nrf2 target genes was verified by qPCR confirming induction ranging from 1.5- to 10-fold for most genes in at least in one PZ-treated HLC (Supplementary Fig. 10).

The degree of coordinated presence of an OS signature in PZ-treated HLCs, was quantified using GSEA which confirmed the ‘OS up’ signature (genes that are induced upon OS, Supplementary Table 5) was significantly enriched in HT1 (p = 0.05) and HT2 (p = 0.02) treated with PZ (Fig. 4d). Similar treatment with PZ produced a less significant enrichment of ‘OS up’ signature in HT3 (p = 0.21), NHT1 (p = 0.22) and NHT2 (p = 0.08). This suggest that PZ-induced OS is detectable from gene expression changes and is similar to a hepatocyte-relevant signature generated by other oxidative stressor drugs. The degree of enrichment of OS signature alone does not directly correspond to the susceptibility phenotype of HLCs, being present in all HLCs.

### Differential transcriptional regulation of iron metabolism genes and iron accumulation in susceptible HLCs

**Figure 5:**
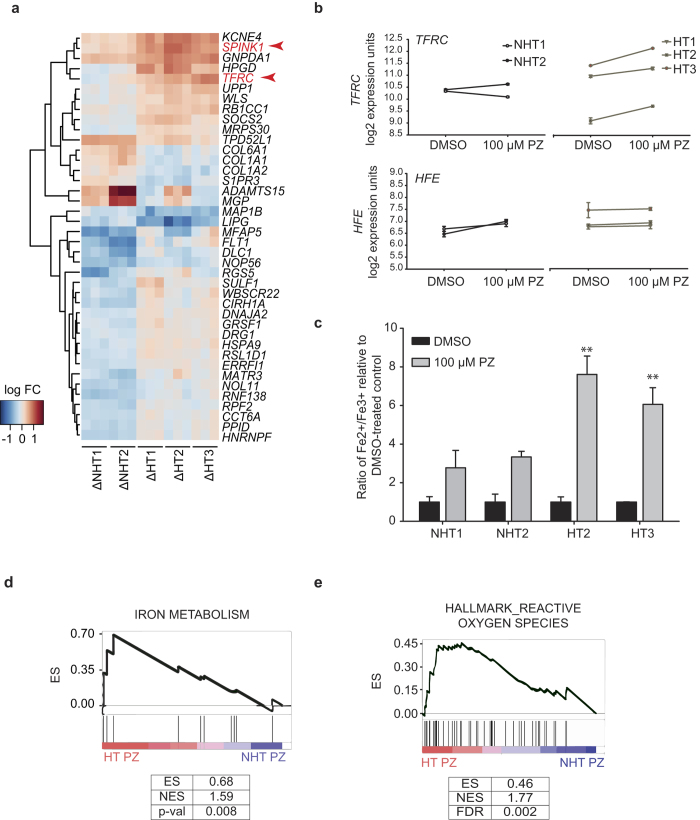
Differential modulation of iron-regulating transcripts and iron metabolism in susceptible HLCs. (**a**) Heatmap of expression of genes in HLCs identified by SAM as being significantly different in HT- and NHT-HLCs after 24 hours treatment with 100 μM PZ. For visualization, the individual log2 fold-changes (log FC) of the top 40 genes was calculated by normalizing expression in PZ-treated samples to the respective vehicle control (DMSO) to yield a baseline-corrected expression represented by Δ. Transcripts highlighted in red *TFRC* and *SPINK1* are related to iron metabolism. (**b**) Expression of *TRFC* mRNA in HLCs showing strong induction of *TFRC* in HT-HLCs (HT1, HT2, HT3) treated with 100 μM PZ for 24 hours. *HFE* mRNA in HLCs with inverse pattern of expression as for *TFRC* with increase in expression seen only for NHT1 and NHT2-HLCs following exposure to PZ. (**c**) Quantification of intracellular iron content in HLCs treated with 100 μM PZ or DMSO for 24 hours. An increase in the Fe^2+^/Fe^3+^ ratio relative to control indicates an accumulation of the redox-active form of iron (Fe^2+^) in cells treated with PZ. Data represents mean ± SE from three independent experiments; **p < 0.001 comparing HT-HLCs to NHT-HLCs treated with PZ. (**d**) GSEA for a custom gene set corresponding iron metabolism genes. (**e**) GSEA for Hallmark gene set for reactive oxygen species from MsigDB. In (**d** and **e**) coordinated upregulation of member genes in gene sets in HT-PZ relative to NHT-PZ samples, is shown. The ES, normalized enrichment score (NES), p-value (for custom gene set) and false discovery rate-adjusted q value (FDR) are indicated for each gene set.

We reasoned that the transcriptional signals of large-scale primary effects of PZ (clearly identifiable in each HLC over its baseline) may mask the differential effects of PZ on HT- and NHT-HLCs. As such, we directly identified genes that are most distinct between HT- and NHT-HLCs treated with PZ by comparing the baseline-normalized expression data (Supplementary Table 6). One of the genes exclusively induced in HT-HLCs was *TFRC* which was on average 1.53-fold higher (p = 4.18E-05) in HT-HLCs compared to NHT-HLCs after PZ treatment (Fig. 5a and Supplementary Table 6). *TFRC* codes for the transferrin receptor protein which mediates the cellular uptake of transferrin-bound iron via endocytosis, and its expression was significantly increased only in HT-HLCs after PZ treatment (Fig. 5b). Conversely, the transcript for *HFE*, the function of which is to limit iron uptake through its interaction with TFRC at the surface of cells^50^ and polymorphisms in which have been linked to PZ-induced serum ALT elevation^31^, showed an increase only in NHT-HLCs after PZ treatment (Fig. 5b). This suggests that in HT-HLCs the modulation of *TFRC* and *HFE* expression can result in a higher intracellular content of iron, potentially exacerbating OS through the generation of highly reactive hydroxyl (^·^OH) radicals from H_2_O_2_ catalyzed by the redox-active form of iron (Fe^2+^) in a ‘Fenton’ reaction^51^. Further, *SPINK1* which was 1.45 times enriched in HT-HLCs (Fig. 5a) is known to be highly expressed in hereditary hemochromatosis-background hepatocellular carcinoma^52^, the genetic background to which is also *HFE* loss. To verify the perturbation of iron levels suggested by the transcriptional profiles of HLCs, we quantified intracellular iron content following exposure to 100 μM PZ and determined the ratio of Fe^2+^ (the redox active form of iron) to Fe^3+^ relative to control-treated cells (Fig. 5c). PZ induced an increase in the ratio of Fe^2+^/Fe^3+^ in all HLCs, however the increase was significantly greater in HT-HLCs at 6- to 7.6-fold compared to 2.8- to 3.3-fold in NHT-HLCs (p = 0.001) (Fig. 5c). Therefore, concomitant to the aberrance in expression of iron regulating genes, we confirmed the greater accumulation of redox-active iron in HT-HLCs.

We examined the expression of a set of genes involved in iron uptake, export and homeostasis (Supplementary Fig. 11), using GSEA and found the coordinated enrichment of iron metabolism in HT-HLCs treated with PZ (p = 0.008) over NHT-HLCs (Fig. 5d). Extending GSEA to curated gene sets from MSigDB^47^ showed a strong enrichment of gene set for ‘reactive oxygen species’ in HT-HLCs (FDR = 0.002) (Fig. 5e) Enrichment was also seen for gene sets for ‘oxidative phosphorylation’, ‘xenobiotic metabolism’, ‘linoleic acid metabolism’, and among KEGG pathways the strongest enrichment observed was for RNA-related processes in the HT group (Supplementary Fig. 12 and Supplementary Table 7). These reflect the differential effects induced by PZ in HT-HLCs with respect to mitochondria function, which is exquisitely sensitive to cellular redox status, and on lipid and RNA functions, which are the likeliest targets of cellular oxidative damage^53^,^54^, particularly that caused by ^·^OH radicals. This suggests that while the primary general effect of PZ induces oxidative stress in HLCs, the burden of this stress is likely higher in susceptible HLCs over the time frame of the cytotoxicity assays, potentially contributed to by the differential regulation of iron metabolism. Therefore, utilizing patient-derived HLCs and gene expression changes we were able to obtain insight into a potentially significant mode of PZ-induced toxicity to hepatocytes centering on OS induced damage.

### PZ induces glutathione depletion and generation of reactive oxygen species in HLCs

**Figure 6:**
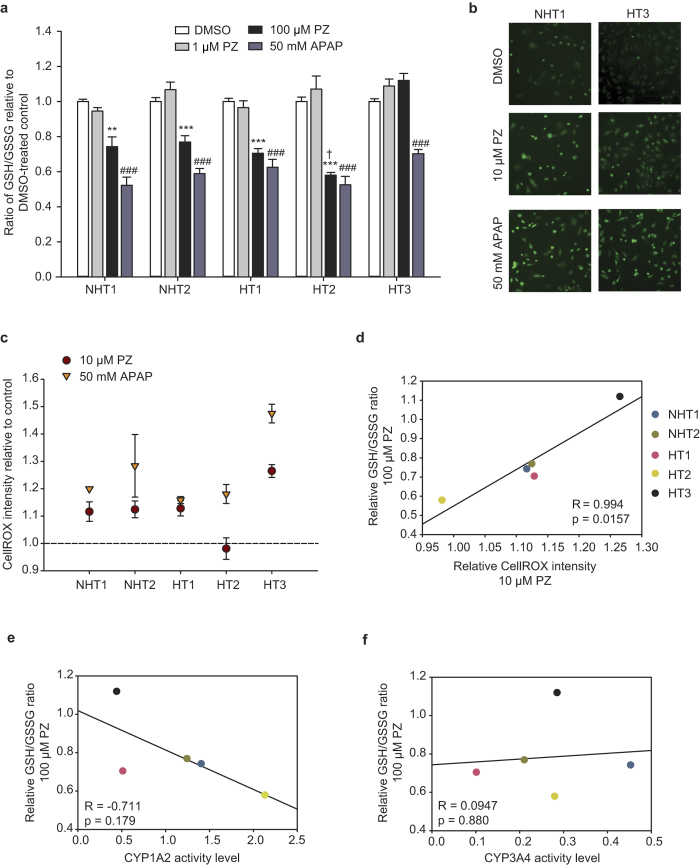
PZ induces early glutathione depletion and accumulation of reactive oxygen species that correlates with CYP1A2 activity. (**a**) Glutathione depletion in HLCs as measured by the ratio of glutathione (GSH) to oxidized glutathione (GSSG) after treatment with pazopanib (PZ) or acetaminophen (APAP) at indicated concentrations for 4 hours. A reduction in GSH/GSSG ratio with respect to control indicates a depletion of GSH or its conversion to GSSG in the treatment group. (**b–c**) Reactive oxygen species (ROS) accumulation in HLCs was measured by CellROX Green fluorescence. HLCs were treated with indicated concentrations of PZ or APAP for 4 hours and in the final hour of treatment 10 μM CellROX Green was supplemented to the medium. (**b**) Representative images of CellROX fluorescence in NHT1 and HT3 showing higher levels of intracellular ROS in HLCs treated with 10 μM PZ and 50 mM APAP compared to control, particularly increased nuclear staining of CellROX. Magnification 100×. (**c**) Quantification of CellROX intensity by flow cytometry where mean fluorescent intensity was measured relative to vehicle control-treated cells for each HLC. **(d)** After 4 hours, relative ROS accumulation (from (c)) is correlated with relative GSH/GSSG ratio (from (**a**)) in HLCs treated with PZ. Greater depletion of GSH, indicated by a lower GSH/GSSG is accompanied by lesser ROS accumulation in HLCs. (**e**,**f**) Correlation of PZ-induced GSH depletion and basal activity of PZ-metabolizing CYPs in HLCs, as previously determined in Fig. 2c and d. Relative GSH/GSSG ratio is inversely correlated with basal CYP1A2 activity levels **(e)** but not with CYP3A4 activity levels **(f).** For (**a**) data represents mean ± SE from three independent experiments in HLCs from differentiation initiated at 3 different times; **p < 0.001 comparing PZ vs. DMSO control; ***p < 0.0001 comparing PZ vs. DMSO control; ^###^p < 0.0001 comparing APAP vs. DMSO control; ^†^p < 0.05 comparing HT2 vs. NHT1 and NHT2-HLCs treated with PZ. For **c.** data represents mean ± SE from two independent experiments. For (**d–f**) each data point represents an individual HLC, indicated by same color legend in (**d**) Correlation coefficient, R and p-values are determined by Pearson’s correlation.

The transcriptionally inferred induction of OS, was further tested by direct cellular measures of OS in HLCs. Drug-induced OS is typically initiated by a drug reactive metabolite (RM), that is produced via the activity of CYP450s. RMs being electrophilic undergo reactions with cellular glutathione (GSH) via chemical or enzymatic-mediated processes^55^, converting GSH to oxidized glutathione (GSSG). To measure the direct OS load generated by PZ reactive metabolite, we measured GSH depletion in HLCs four hours after drug treatment when secondary effects were minimal. At high dose of 100 μM, PZ caused a significant depletion of GSH in all HLCs (p < 0.001), except HT3 (Fig. 6a). When treated with 50 mM APAP as a positive control, significant GSH depletion was also evident for all HLCs, in line with the known glutathione-conjugating effect of the APAP reactive metabolite, N-acetyl-p-benzoquinone imine (NAPQI) (p < 0.0001) (Fig. 6a). N-acetyl cysteine (NAC), which serves as precursors to GSH synthesis, could rescue the viability of HT1- and HT2-HLCs when co-incubated with PZ (Supplementary Fig. 13). In all, the results suggest that PZ initiates early GSH depletion in 4 out of 5 HLCs, which is consistent with the formation of a RM.

As GSH is required for the function of several redox regulating enzymes, an alteration to its levels can lead to the disruption of the redox balance of the cell and allow reactive oxygen species (ROS) such as O_2_^·−^ and H_2_O_2_, produced in cellular components like mitochondria to accumulate. Therefore, we looked for evidence of ROS accumulation in HLCs treated with PZ. Treated cells were exposed to the fluorogenic probe, CellROX Green, the fluorescence of which increases upon oxidation by ROS. Relative to control, CellROX fluorescence intensity increased 1.11- to 1.26-fold in all HLCs, except HT2, when treated with 1–10 μM PZ for 4 hours (Fig. 6b and c, Supplementary Fig. 14). At 100 μM, we observed interference of the CellROX fluorescence signal with possible autofluorescence from PZ (data not shown). In control experiments, 50 mM APAP treatment for 4 hours, generated 1.16- to 1.48-fold increase in CellROX intensity (Fig. 6b and c). We noted that CellROX intensity with 10 μM PZ was strongly correlated to the GSH/GSSG ratio, representing GSH depletion (R = 0.994; p = 0.0157) in HLCs (Fig. 6d). This suggested that at 4 hours after PZ treatment, GSH likely acts as direct scavenger of ROS and is removed from the cellular pool upon its oxidation by the transient increase in ROS initiated by PZ^56^. Importantly, this data accounts for the apparent lack of accumulation of ROS in HT2 (Fig. 6b), and the lack of GSH depletion in HT3 (Fig. 6a), as each occupies an extreme position on the correlation plot (Fig. 6d). Notwithstanding the kinetics of variations in ROS and GSH amounts, it is apparent that incubation of HLCs with PZ leads to perturbation of their redox state, as reflected by both GSH depletion and ROS accumulation. A similar correlation between GSH depletion by 50 mM APAP and CellROX intensity in HLCs was also evident (Supplementary Fig. 15a), suggesting there is an inverse relationship between the cellular GSH pool and the accumulation of ROS, for both PZ and APAP, appearing to be a general effect of a hepatotoxic drug on HLCs. In all, at 4 hours, HT3 appeared most prone to accumulation of ROS and attendant lack of GSH depletion, while HT2 had the greatest GSH depletion and least ROS accumulation (for both PZ and APAP).

Putting the metabolic activity of HLCs in context with the above observations, we show that the degree of GSH depletion by treatment with 100 μM PZ is correlated with basal CYP1A2 activity of individual HLCs (R = −0.711), but not with their CYP3A4 activity (R = 0.0947) (Fig. 6e and f). Similar correlation with CYP1A2 activity was also apparent for GSH depletion induced with 50 mM APAP (Supplementary Fig. 15b and c). This strongly suggested that in patient-specific HLCs the metabolic activity of CYP1A2 was the major contributor to the formation of putative RM (indirectly measured as GSH depletion over 4 hours), both for PZ and APAP. However, at least *in vitro,* CYP1A2 activity levels were not distinguishable between HT and NHT groups, suggesting additional underlying patient-specific features act on the initial OS-inducing effect of PZ to elevate it to phenotypically distinct and measurable levels.

In support of the theory that factors not related to the primary metabolism of PZ by CYPs play a role in the increased OS and sensitivity in certain patient-derived HLCs, we measured intracellular parental PZ concentration in HLC lines 4 hours after exposure to 100 μM PZ. Using Liquid chromatography-Mass Spectrometry (LC-MS), we show that parental PZ accumulates to highly comparable amounts in all HLC lines (Supplementary Fig. 16). This furthers the notion that differential metabolism and intracellular accumulation alone cannot account for differential cytotoxic effects of PZ.

Genotyping of individuals for polymorphisms of CYPs relevant to PZ metabolism revealed the occurrence of the CC/CA allele for *CYP1A2* rs762551 in the HT cases, and AA allele in NHT cases (Supplementary Table 8). This suggests variants of *CYP1A2* are distinct in HT- and NHT-HLCs and could be contributing factors in a patient’s susceptibility to PZ, over the longer course of drug administration clinically. Additionally, we examined polymorphisms in genes related to pharmacokinetics and pharmacodynamics of PZ and for antioxidant defense to examine further contribution of distinguishing variants to patient variability in hepatotoxicity. HT cases had specific variants for the *ABCB1* (mediator of PZ efflux) and *VEGFR2* (pharmacological target of PZ) (Supplementary Table 9). Taken together, our results indicate that PZ-mediated hepatotoxicity is initiated by the drug’s intrinsic property of inducing OS which is correlated *in vitro* to CYP1A2 activity measurable as immediate effects. The distinction between susceptibility phenotypes could not be simply attributed to OS induced by reactive metabolites or the contribution of CYP activity in HLCs. The combination of measurable differences in iron metabolism and genetic polymorphisms identified through the comparison of HT and NHT-HLCs are candidates for patient risk factors to PZ-induced hepatotoxicity.

## Discussion

Being genetic matches to individuals with distinct clinical phenotypes, iPSCs have been applied for the modeling of several monogenic diseases with well-defined causative mutations^57^, and for drug-induced toxicities with clearly associated genetic variations or mutations^20^,^58^. In this study, we further the application of genetically-matched iPSCs and demonstrate for the first time their utility in modeling drug-induced idiosyncratic toxicities, which result from potentially multiple uncharacterized predisposing genetic factors harbored by patients. We show that patient iPSC-derived hepatocyte-like cells (HLCs) could successfully model adverse drug reactions to pazopanib (PZ), a drug with clinically relevant hepatotoxicity, and delineate the multi-factorial mechanistic nature of its toxicity.

This work relied heavily on a robust system to generate HLCs from pluripotent stem cells. A relatively facile system for generating iPSCs based on non-integrating episomal method for reprogramming immortalized lymphocytes was adopted^33^,^34^ and for each iPSC line, a similar and high degree of differentiation to HLCs with liver-specific functions, including albumin production and CYP activities were achieved^35^. Being indistinguishable in their overall hepatic functions, the HLCs were suitable for studying patient-specific drug-induced hepatotoxicity (requiring drug metabolism)^59^. As exemplified with PZ, the successful generation of functional iPSC-derived HLCs essentially provides an unlimited supply of patient-specific cells to perform transcriptional profiling and diverse cellular assays to shed light on the multi-factorial mechanism of PZ hepatotoxicity, which has remained uncertain despite the high rate of associated clinical hepatotoxicity^28^. We believe this approach to studying idiosyncratic reactions is generally applicable to several hepatotoxic drugs of unknown mechanistic basis.

To our knowledge, this is the first demonstration on the application of toxicogenomics analyses on patient-derived hepatocytes, which has so far being limited to primary hepatocytes^60^,^61^,^62^,^63^,^64^, to seek differences among individuals that may contribute predisposing features for hepatotoxicity. Using GSEA to obtain a meaningful overview of the cellular mechanisms affected by PZ, we found that perturbations to lipid and RNA metabolism occur in HT-HLCs within the context of differential modulation of oxidative phosphorylation and reactive oxygen species pathways. We identified a potential role for altered iron metabolism in exacerbating redox imbalance in HT-HLCs through the modulation of transcript levels of *TFRC* and *HFE*. This finding is particularly relevant, as an association between the *HFE* polymorphism rs2858996, which has been predicted to reduce its expression compared to wildtype allele, and PZ-induced hepatic dysfunction has been documented^31^. The report suggested that PZ-mediated hepatotoxicity might result from the pharmacological inhibition of its targets including VEGFRs, since HFE induction is suppressed when VEGF signaling is inhibited^31^. Our study has independently implicated altered iron metabolism in PZ-mediated hepatotoxicity, quantifiable as an increase in redox-active iron in HLCs, and generated an alternative hypothesis that altered iron metabolism exacerbates the oxidative stress load produced by PZ and its reactive metabolites.

The generation of patient-derived HLCs also allowed the recapitulation of cellular responses to PZ-induced toxicity. Even in NHT-HLCs and primary human hepatocytes, PZ reduces viability to ~70% compared to untreated cells, suggesting an inherent property of PZ induces damage in hepatocytes. First through transcriptional analysis and then through cellular measures, we show a generalized GSH depletion and ROS accumulation in HLCs within four hours of PZ administration. This demonstration made for the first time in HLCs for PZ, agrees with the evidence of RM formation from PZ in microsomal assays, that show time-dependent inhibition of CYP3A activity and the trapping of reactive intermediates of PZ by glutathione^65^. Although circulating RM have not been identified yet for PZ, future efforts on this front would be of interest in the light of our data and the background knowledge that a number of other TKIs which share physicochemical and metabolism properties with PZ are metabolized to reactive intermediates^29^. Further, the relatively high dose of PZ administration compared to other TKIs makes the potential consequences of RM formation even more relevant^66^.

In placing our results in metabolic context, we found that all three HT patients were carriers of similar germline variants (CC/CA) for *CYP1A2* rs762551, for which the ‘C’ allele has been associated with adverse cardiac effects from chlorpromazine resulting from increased plasma drug exposure^67^. No association has previously been made with *CYP1A2* polymorphisms and clinical hepatotoxicity in RCC patients on PZ therapy^32^. There may be differences arising from ethnicity as polymorphic association studies have been conducted primarily in Caucasians^31^,^32^ and all five patients in the present study were of East Asian origin. Interestingly, among the five HLCs studied here, we did find a correlation between basal CYP1A2 activity and GSH depletion from PZ treatment. Several lines of evidence therefore implicate CYP1A2 in the metabolism of PZ to potentially reactive intermediates. Additionally, we also found the specific ‘TT’ variant for *ABCB1* rs2032582 in HT cases. PZ is a substrate of *ABCB1* which mediates its efflux, however there is no concordant outcome related to drug exposure and drug effect for this *ABCB1* polymorphism^68^.

In conclusion, despite the above polymorphic observations, we could not ascribe any one genetic feature as a risk factor for PZ–mediated hepatotoxicity. This reinforces our assertion that unlike disease phenotypes that can be correlated to a single genetic variant previously modeled with iPSC-derived cells^69^, idiosyncratic hepatotoxicity has multiple causative factors, and making associations with single risk factors inevitably has its pitfalls^23^. Hence, we advocate the use of patient-derived HLCs for the assessment of drug and patient features in their totality, with the application of combined measures of cellular viability, oxidative stress and gene expression as readouts for quantifying degrees of hepatotoxicity.

Given the relatively small sample size for this work (5 patients), the utilization of any given degree of differential cellular sensitivity (e.g.~25% above control lines) as a readout for potential hepatotoxicity may not represent full statistical rigor. We acknowledge this sample size limitation, which would relate to the concern of whether inter-individual variability may account for the differential observations between the 3 cases and 2 controls. Nonetheless, the concordance between clinical observations and multiple (in contrast to single) cellular phenotypic assays - including viability, oxidative stress and iron levels- reduces the possibility of chance arrival at conclusion of differential effects of PZ and supports true variability measurable in cellular models. The concordance observed between the patient and the cellular phenotypes here may therefore be interpreted as a proof of concept, and a foundation for future studies with expanded patient numbers. Despite statistical limitations from sample size, this work provides early support for investigating patient-specific HLCs in modeling a highly specific idiosyncratic drug reaction, when knowledge of factors underlying such individual hepatotoxicity is scarce. In turn, these models can be further interrogated to determine the potential mechanisms driving such toxicity. Patient-specific HLCs are useful tools for interrogating drug- and patient-specific features that contribute to individual susceptibility. Finally, as parts of cellular banks, characterized HLCs can also be valuable in the context of pharmaceutical drug testing by being representative of population genetic diversity.

## Methods

### *In vitro* differentiation of iPSCs to hepatocyte-like cells (HLCs)

The differentiation of iPSCs into hepatocytes was carried out as described previously^35^ with slight modification. Briefly, iPSCs were cultured to 70% confluence before switching to a basal differentiation medium containing 100 ng/ml activin A (R&D systems) and 50 ng/ml WNT3a (R&D systems) for 6 days (step 1), followed by 10 ng/ml FGF2 (R&D systems) and 50 ng/ml BMP4 (R&D systems) in differentiation medium (half basal differentiation medium with half STEMdiff™ APEL™ medium (Stemcell Technologies)) for 4 days (step 2). On the following 4 days, 50 ng/ml FGF1 (R&D systems), 10 ng/ml FGF4 (R&D systems) and 25 ng/ml FGF8 (R&D systems) was applied in the differentiation medium (step 3). Finally, the cells were incubated with 20 ng/ml HGF (R&D systems) and 100 ng/ml Follistatin-288 (R&D systems) for 4 days with additional 20 ng/ml Oncostatin (R&D systems) for another 2 days (step 4). The medium was changed every other day during the 20-days differentiation period.

### Cytotoxicity measurement

HLCs were harvested after 20 days of hepatic differentiation cells with 2× TrypLE (Life Technologies), and plated onto Matrigel (Corning)-coated 96 well plate at the density of 3 × 10^4^ cells per well for overnight incubation. Cells were then treated with serially diluted concentrations of acetaminophen (APAP, Sigma) and pazopanib hydrochloride (Selleck Chemicals) in the basal differentiation medium supplemented with 20 ng/ml HGF (R&D systems), 100 ng/ml Follistatin-288 (R&D systems) and 20 ng/ml Oncostatin (R&D systems) for 24 hours. Drug dilutions were prepared in DMSO. Cell viability was determined by the MTS assay using CellTiter 96^®^ AQueous One Solution Cell Proliferation Assay kit (Promega) and the ATP assay was performed with CellTiter-Glo^®^ Luminescent Cell Viability Assay kit (Promega).

For viability experiments with N-acetyl cysteine (NAC), a similar procedure was adopted except cells were co-incubated with 0.5 mM NAC (Hidonac^®^, Zambon S.p.A.) and PZ for 24 hours. Vehicle controls were appropriately modified to include 0.05% EDTA in PBS (in which NAC was dissolved). Cell viability was measured using CellTiter-Glo reagent.

### Cryopreserved primary human adult hepatocytes

Cryopreserved primary human adult hepatocytes were purchased from vendors including Life Technologies (Gibco HP4239, HP4248, Lot 4227 and Lot 8105) and BD Biosciences (Lot 246). Human hepatocytes were thawed and pelleted by centrifugation at 50 g for 5 minutes at 4 °C. For RT-PCR assay, the cell pellet was lysed in Buffer RLT Plus (Qiagen) and stored at −80 °C for future processing. For hepatic function assay, the pellet was resuspended in hepatocyte culture medium and further seeded on Type I bovine collagen (Advanced BioMatrix) coated tissue culture plate.

### Transcriptional analysis of HLCs using microarrays

For expression analysis to study PZ-induced transcriptional changes, all five HLC lines were treated with 100 μM PZ or DMSO for 24 hours. For each group, RNA from three biological replicates was isolated using RNeasy Plus Micro kit (Qiagen), except for HT2-DMSO group for which duplicates were used. A total of 29 samples were analyzed. Preparation of samples for microarray hybridization and scanning was done at Biopolis Shared Facilities, Singapore. The Affymetrix Human Genome U133 Plus 2.0 platform was used. CEL files were imported and normalized using *rma* from *affy* package in R version 3.2.1. Data was further analyzed using package *genefilter* to filter microarray data to retain features detectable in at least five samples (out of 29 samples) with log2 scale expression value greater than 9 (6700 probes). Differentially expressed genes were identified using SAM function of *siggenes* package on the filtered dataset by comparing PZ-treated samples to DMSO controls, labeled as PZ or control. To select genes that were differentially expressed between HT-HLCs and NHT-HLCs after PZ treatment, SAM was applied on control-normalized expression data i.e. normalized to respective DMSO controls and appropriate labels were applied for HT and NHT samples. Heatmaps of gene expression were drawn using heatmap.2 function of *gplots* package in R. Principal component analysis (PCA) was done using selected features and PCA plots were generated by isolating and first and second principal components and plotting them on x- and y-axes.

### Quantification of glutathione/oxidized glutathione and reactive oxygen species (ROS)

Cellular glutathione and oxidized glutathione content was measured in 96-well plates using GSH/GSSH-Glo assay (Promega). HLCs were harvested and seeded at 3 × 10^4^ cells per well of Matrigel-coated plate, allowed to adhere overnight, and then treated with drug or vehicle control for 4 hours before cell lysis to measure relative amounts of total and oxidized glutathione. Ratio of glutathione and oxidized glutathione was calculated from net relative light units (RLU, after background subtraction) as recommended in the manufacturer protocol. For the quantification of ROS, the CellROX Green reagent was used (Molecular Probes, Life Technologies). HLCs were seeded in Matrigel-coated 24-well plates at the density of 1 × 10^4^ cells per well and allowed to adhere overnight. HLCs were incubated with PZ at indicated concentrations or with vehicle (DMSO) for a total of 4 hours. In the final hour i.e. 3 hours after drug incubation, 10 μM CellROX Green was added. Cells were washed with PBS, collected by trypsinization, resuspended in 0.5% BSA in PBS for analysis by flow cytometry for green fluorescence and 50,000 events per sample were collected. For imaging of CellROX fluorescence, cells were washed with PBS following incubation with 10 μM CellROX Green and imaged with a fluorescence microscope (IX81 Olympus).

### Intracellular iron measurement

Measurement of intracellular iron concentration was done using the Iron Colorimetric Assay Kit (Biovision), as per the manufacturer’s instructions with modifications. HLCs were seeded in Matrigel-coated 12-well plates, at density of 500,000–800,000 cells/well and allowed to attach overnight, following which HLCs were treated with 100 μM PZ or DMSO as control. Twenty four hours after treatment, HLCs were washed with PBS and detached using Accutase. HLCs were centrifuged and washed and finally resuspended in 100 μl of Iron Assay Buffer, following which the harvested cell count was determined. Cell lysis did not occur in the Iron Assay Buffer, therefore to induce lysis 5 μl of 1 M SDS was added and cell suspension was thoroughly vortexed. Cellular debris was removed by centrifugation for 10 minutes at 16,000 × g. For determination of total (Fe^2+^ and Fe^3+^) and reduced (Fe^2+^) iron amounts separately, 100 μl lysate was split two-ways to give a sample volume of 50 μl per measurement. Colorimetric measurement of iron concentration was done as per instructions and absorbance was measured at wavelength of 593 nm and converted to nmol amount based on standard curve that was included in each run. Finally, the amounts of Fe^2+^ and Fe^3+^ (as inferred from the total and Fe^2+^ amounts) in nmol was normalized per cell using cell count determined prior to lysis. For each HLC line, ratio of Fe^2+^ to Fe^3+^ was determined based on cell number-normalized concentration measures for control- and PZ-treated samples.

### Statistical Analysis

Data are presented as the mean ± s.e.m. Statistical significance was determined by unpaired Student’s t-test and two-tailed p value of <0.05 was considered to be statistically significant. Correlation analysis was done using Pearson’s correlation analysis.

### Clinical Samples

Informed consent was received from all subjects prior to inclusion in study. All experimental protocols involving human subjects, including generation of iPSCs from peripheral blood mononuclear cells, were approved by the SingHealth Centralised Institutional Review Board (Protocol #MMHPC-2011). All methods were carried out in accordance with the approved guidelines.

### Animal experiments

For animal studies, all experimental protocols were approved by the National Advisory Committee for Laboratory Animal Research, Singapore (Protocol #130891). All methods were conducted in accordance with the approved Guidelines on the Care and Use of Animals for Scientific Purposes.

## Additional Information

**Accession numbers**: Microarray data described herein have been deposited in NCBI Gene Expression Omnibus (http://www.ncbi.nlm.nih.gov/geo/), with accession number GSE75888, in a MIAME-compliant format.

**How to cite this article**: Choudhury, Y. *et al*. Patient-specific hepatocyte-like cells derived from induced pluripotent stem cells model pazopanib-mediated hepatotoxicity. *Sci. Rep.* **7**, 41238; doi: 10.1038/srep41238 (2017).

**Publisher's note:** Springer Nature remains neutral with regard to jurisdictional claims in published maps and institutional affiliations.

## Supplementary Material

Supplementary Data
