# Corrigendum: Patient-specific hepatocyte-like cells derived from induced pluripotent stem cells model pazopanib-mediated hepatotoxicity

**DOI:** 10.1038/srep46391

**Published:** 2017-04-25

**Authors:** Yukti Choudhury, Yi Chin Toh, Jiangwa Xing, Yinghua Qu, Jonathan Poh, Huan Li, Hui Shan Tan, Ravindran Kanesvaran, Hanry Yu, Min-Han Tan

Scientific Reports 7: Article number: 4123810.1038/srep41238; published online: 01 25 2017; updated: 04 25 2017

The original version of this Article contained an error in the spelling of the author Huan Li, which was incorrectly given as Li Huan.

The Author Contributions Statement,

Study concept and design: Y.C., Y.C.T., M.H.T. Acquisition of data: Y.C., Y.C.T., Y.H.Q., J.W.X., L.H., J.P., H.S.T., R.K., H.Y., M.H.T. Analysis and interpretation of data: Y.C., Y.C.T., Y.H.Q., J.W.X., L.H., J.P., M.H.T. Manuscript drafting: Y.C., Y.C.T., Y.H.Q., M.H.T. Critical revision of manuscript: Y.C., Y.C.T., Y.H.Q., J.W.X., L.H., J.P., H.S.T., R.K., H.Y., M.H.T. Statistical analysis: Y.C., Y.C.T., J.W.X., L.H., Y.H.Q., J.P. Obtaining funding: H.Y., M.H.T.

now reads:

Study concept and design: Y.C., Y.C.T., M.H.T. Acquisition of data: Y.C., Y.C.T., Y.H.Q., J.W.X., H.L., J.P., H.S.T., R.K., H.Y., M.H.T. Analysis and interpretation of data: Y.C., Y.C.T., Y.H.Q., J.W.X., H.L., J.P., M.H.T. Manuscript drafting: Y.C., Y.C.T., Y.H.Q., M.H.T. Critical revision of manuscript: Y.C., Y.C.T., Y.H.Q., J.W.X., H.L., J.P., H.S.T., R.K., H.Y., M.H.T. Statistical analysis: Y.C., Y.C.T., J.W.X., H.L., Y.H.Q., J.P. Obtaining funding: H.Y., M.H.T.

In addition, the Acknowledgements section in this Article was incomplete.

“This work is supported by Institute of Bioengineering and Nanotechnology, BMRC & JCO-DP, Agency for Science, Technology and Research in Singapore (A*STAR), and Janssen. JWX was an MBI PhD scholar. We would like to thank Dr. Johnathan Lai and Ms Foong Yu Miin for generating EBVi cell lines used in this study, and Prof Chan Soh Ha for kindly providing B95–8 cells for the production of EBV. We acknowledge the helpful services of Dr. Parul Saxena and Dr. Wen Chaoming from Biopolis Shared Facilities, A*STAR, for performing the Affymetrix microarray experiments.”

now reads:

“This work is supported by Institute of Bioengineering and Nanotechnology, BMRC & JCO-DP, Agency for Science, Technology and Research in Singapore (A*STAR), and Janssen. JWX was an MBI PhD scholar. We would like to thank Dr. Johnathan Lai and Ms Foong Yu Miin for generating EBVi cell lines used in this study, and Prof Chan Soh Ha for kindly providing B95–8 cells for the production of EBV. We would like to thank Dr. Joanne Ngeow for administrative support rendered. We acknowledge the helpful services of Dr. Parul Saxena and Dr. Wen Chaoming from Biopolis Shared Facilities, A*STAR, for performing the Affymetrix microarray experiments.”

