# Supplementary material for: Patient-specific hepatocyte-like cells derived from induced pluripotent stem cells model pazopanib-mediated hepatotoxicity
Source: Sci Rep. 2017 Jan 25;7:41238. doi: 10.1038/srep41238 (PMC5264611; doi:10.1038/srep41238)
Supplement: Supplementary Data [file srep41238-s1.pdf]

SUPPLEMENTARY INFORMATION

Patient-specific hepatocyte-like cells derived from induced pluripotent stem cells model pazopanib-mediated hepatotoxicity

Yukti Choudhury[1,†], Yi Chin Toh[1,2,†], Jiangwa Xing[1], Yinghua Qu[1,#], Jonathan Poh[1], Li Huan[1], Hui Shan Tan[3], Ravindran Kanesvaran[3], Hanry Yu[1,4,5‡], Min-Han Tan[1,3,‡,*]

[1] Institute of Bioengineering and Nanotechnology, 31 Biopolis Way #04-01, The Nanos, Singapore 138669, Republic of Singapore
[2] Department of Biomedical Engineering, Faculty of Engineering, National University of Singapore, 4, Engineering Drive 3, E4 #04-08, Singapore 117583, Republic of Singapore
[3] Division of Medical Oncology, National Cancer Centre, Singapore 169610, Republic of Singapore
[4] Yong Loo Lin School of Medicine and Mechanobiology Institute, National University of Singapore, Republic of Singapore
[5] Gastroenterology Department, Nanfang Hospital, Southern Medical University, Guangzhou 510515, China

Corresponding author:

Min-Han Tan, MBBS, MRCP, PhD

Principal Research Scientist and Team Leader, Institute of Bioengineering and Nanotechnology

31 Biopolis Way, #04-01, Singapore 138669, Republic of Singapore

T: (65) 6824 7110

F: (65) 6478 9080

E: mhtan@ibn.a-star.edu.sg

SUPPLEMENTARY MATERIALS AND METHODS

Epstein-Barr Virus (EBV) immortalization of patient lymphocytes (EBVi cells)

For creating EBV immortalized patient cell lines, peripheral blood mononuclear cells (PBMCs) were isolated from peripheral blood using Ficoll-Paque. The cell pellet was washed with PBS and resuspended in 2 ml of medium for EBV infection (RPMI1640 with HEPES, 20% FBS, 1% penicillin-streptomycin and 0.2 µg/ml cyclosporine-A), to which 1 ml of EBV-containing medium from B95-8 cell lines was added [1]. The cells were incubated for at least 48 hours in a 37°C/5% CO_2 incubator. Following this, cells were washed and resuspended in fresh medium and allowed to grow with half medium change every 7 days. After two to three weeks of EBV infection, cell clusters were visible which were then maintained as EBVi cell lines. All EBVi cell lines were maintained in RPMI1640 with 20% FBS at 37°C and 5% CO2 in a humidified incubator as suspension cultures.

Derivation of iPSCs from EBV-immortalized B-lymphocytes

Derivation of iPSCs from EBVi cell lines was essentially done as described in the past for human lymphoblastoid lines, with some modifications[2]. EBVi lines from 5 patients HT1, HT2, HT3, NHT1, NHT2 were reprogrammed to patient-specific iPSCs. Two million EBVi cells were transfected with 2 µg each of two EBNA-1/OriP-based episomal vectors encoding reprogramming factors. Nucleofection was done with Amaxa nucleofector device II (Lonza) with the programme X-001. After nucleofection, cells were transferred to RPMI medium and allowed to recover for 12-16 hours following which they were transferred to one well of a Matrigel-coated 6-well plate in reprogramming medium. The components of reprogramming medium are DMEM/F12 supplemented with N-2, B-27, non-essential amino acids, Glutamax, 0.1 mM 2-mercaptoethanol, 100 ng/mL human bFGF (Invitrogen), 0.5 µM PD0325901, 3 µM CHIR99021, 0.5 µM A-83-01 (Stemgent), 1000 units/mL human LIF (Millipore) and 10 µM HA-100 (Santa Cruz). Reprogramming medium was replaced every two days, while ensuring minimal loss of cells in suspension during the first two weeks of culture, maximally allowing cells to transition to adherent morphology. Around day 15 when a large number of cells had adhered to the matrigel, the population of floating cells was removed from culture and adherent cells were allowed to form colonies. Depending on EBVi cell line, large colonies resembling reprogrammed iPSCs appeared between days 35-53 from initial nucleofection. Colonies were picked and transferred to 48-well plates and over one week transitioned to mTESR1 medium. True iPSC colonies were verified by morphology and live-staining of TRA-1-60 antibody. Live staining of iPSCs for TRA-1-60 detection was done using StainAlive TRA-1-60 Antibody conjugated to DyLight™ 488 (Stemgent), following recommended protocol. Briefly, medium was replaced with medium containing antibody diluted 1:100 and cells were incubated for 30 minutes, following which they were washed once, the medium replaced and imaged. Selected iPSC colonies were propagated using mTESR1 medium on matrigel-coated plates. Culture of derived iPSCs was done on matrigel-coated plates with mTESR1 medium (Stemcell Technologies). Routine passage of iPSC lines was done using

Dispase (Stemcell Technologies) and iPSCs of passage numbers fewer than 30 were used for all experiments.

iPSC characterization

Total RNA from EBVi cells, iPSCs and embryoid bodies was extracted using the Rneasy Mini Plus kit (Qiagen). Three to 5 µg RNA was subjected to Turbo DNAse treatment (Ambion) to remove contaminating genomic DNA. One microgram of DNAse-treated RNA was converted to cDNA using SuperScript III First-Strand Synthesis System (Invitrogen) with random hexamer primers. Synthesized cDNA was diluted 10-fold before use in PCR reactions using the primers for pluripotency markers listed in "Primers" section of Supplementary Materials and Methods. For the formation of embryoid bodies, confluent iPSCs from one well of 6-well dish were washed, treated with dispase and detached using a cell scraper to form large cell clumps. The cell suspension was collected and transferred to a non-adherent cell culture dish containing stem cell medium. Embryoid bodies (EB) were allowed to form over 7 days in suspension and medium was replaced every two days. Detection of markers of three lineages mesoderm, ectoderm and endoderm in embryoid bodies was done on cDNA prepared from RNA extracted from EBs. The primers used for lineage detection are listed in "Primers" section. PCR was done using 1 µl of 10-fold diluted cDNA in 20 µl PCR reaction with HotStarTaq PCR master mix (Qiagen) for 35 cycles at 94°C for 1 min, 51-55°C for 30 s and 72°C for 30-60s.

For immunostaining of pluripotency markers in iPSCs, cells were fixed with 4 % paraformaldehyde and permeabilized with 0.1% Triton X-100 in PBS. Staining was done with antibodies against OCT3/4 (1:200, sc-5279, Santa Cruz), SSEA-4 (1:200, 90231, Merck Millipore), TRA-1-60 (1:200, 90232, Merck Millipore) and Nanog (1:1000, 48935, Cell Signaling). Signal was detected with secondary antibodies IgM-FITC (sc-2012, Santa Cruz), IgG-FITC (sc-2010, Santa Cruz) and IgG-AlexFluor594 (A-21203, Life Technologies). All secondary antibodies were diluted 1:500. For flow cytometric analysis of iPSCs, single-cell suspension of iPSC cultures was prepared with Accutase (Life Technologies) and cells were fixed in 2% paraformaldehyde. For intracellular antigen OCT4, fixed cells were permeabilized with 0.1% TritonX-100. Incubation with fluorophore conjugated primary antibody against OCT4 (FCMAB113A4, Merck Millipore), or TRA-1-60 (09-0068, Stemgent), or isotype controls was done for 30 mins before analysis with flow cytometry (Beckman Coulter).

For teratoma assay, 3×10^6 iPSCs were mixed with Matrigel in 1:1 volume ratio and injected subcutaneously into NOD-SCID mice. Tumors were excised when they reached 2 cm in diameter 8-10 weeks later and were fixed, paraffin-embedded and processed for hematoxylin and eosin staining at the Core Histopathology Laboratory in Institute of Molecular and Cell Biology at Biopolis, Singapore. All animal experiments were conducted under the Guidelines on the Care and Use of Animals for Scientific Purposes issued by the National Advisory Committee for Laboratory Animal Research, Singapore.

Karyotyping analysis by G-banding was done at the Cytogenetics Laboratory, Department of Pathology at Singapore General Hospital.

Determination of genetic polymorphisms

Genomic DNA was extracted from EBVi or iPSCs cell lines using Dneasy Blood and Tissue kit (Qiagen). PCR to detect polymorphisms was performed using primers listed in “Primers” section. PCR was done using 100 ng of genomic DNA per reaction and HotStarTaq PCR master mix (Qiagen) for 35 cycles at 94°C for 1 min, 51°C for 30 s and 72°C for 30 s. PCR products were resolved on agarose gel and amplicons of appropriate size were subjected to Sanger sequencing with the same primers used for amplification to determine polymorphisms (SNP). The position of each SNP in gene’s context is taken the latest annotation from dbSNP.

Characterization of hepatocyte-like cells (HLCs)

For detection of albumin-positive cells by flow cytometry, after 20 days hepatic differentiation, cells were dissociated with 2×TrypLE (Life Technologies) and passed through cell strainer (Mesh size: 70 μm, Corning Life Sciences) to get single cells. Approximately 1×10^6 cells were then fixed in 3.7% paraformaldehyde for 15 minutes and permeabilized with Triton X-100 0.5% (v/v) for 20 minutes and then blocked in blocking buffer (2% BSA with 0.1% Triton-PBS) overnight. For each analysis, $1\text{-}5\times10^5$ cells were used per sample. Cells were incubated for 1 hour at room temperature with Anti-Human Serum Albumin antibody-FITC (Abcam) or human IgG-FITC isotype control (Santa Cruz).. Cells were then analyzed by a FACS Calibur Flow Cytometer (BD Biosciences) and analyzed using CellQuest software (BD Biosciences).

For measuring urea production, 20 days after hepatic differentiation, cell suspension medium was collected for hepatocyte functionality assay. Urea secretion was evaluated using the Urea Nitrogen Kit (Stanbio Laboratory). Cell number was counted during the following cell harvesting steps, and the number was used to normalize the functional data.

For cytochrome 450 metabolic activity assays, before cells were harvested on day 20 of differentiation, Krebs-Henseleit buffer (KHB, Sigma) with CYP specific probe substrates (CYP1A2: 200 mM phenacetin and CYP3A4: 5 mM midazolam) was added in the respective wells and incubated for 1 hour. Then, the supernatant was collected and the metabolites were measured by chromatography-mass spectrometry (LC/MS). The gained activity data was normalized to the cell number counted during the following cell dissociation steps of the respective well.

Total RNA was isolated from differentiated cells using the RNeasy Plus Micro kit (Qiagen). For each sample, 1 μg of total RNA was reverse-transcribed to cDNA by iScript™ Reverse Transcription Supermix (Bio Rad). RT-PCR was then carried out with FastStart Universal SYBR Green master mix (Roche

Applied Science) and specific primers (GeneCopoeia) as listed in "Primers" section. The reaction was performed using a 7500 Fast Real-Time PCR system (Life Technologies) in duplicates and normalized to *GAPDH* in the same run.

Measurement of intracellular pazopanib content by Liquid Chromatography-Mass Spectrometry

HLCs from different iPSC lines were seeded into 12 well plates at a density of 200,000 cells/well and treated with 100 µM PZ for 4 hr. Cells were harvested with 2×TrypLE, quenched in 1ml cold methanol and then subjected to 3 freeze-thaw cycles using liquid nitrogen and ultrasonication in ice bath for 30 min. Samples were centrifuged and supernatants were collected for PZ content measurement using Liquid Chromatography-Mass Spectrometry (LC-MS Finnigan LCQ Deca XP Max, Agilent 1100 series).

A standard curve of PZ was generated and measured together with PZ-treated samples. PZ content was quantified on Agilent 1100 HPLC (Agilent Corp., Santa Clara, CA, USA) with AB Sciex QTrap 3200 tandem mass spectrometer (AB Sciex, Framingham, MA,USA). The HPLC system was equipped with a binary pump, an online vacuum degasser, an autosampler, and automatic thermostatic column oven. Analytical separation was performed on Phenomenex (4.6 mm X 100 mm, 5 µM) at 25 °C with a flow rate 0.8 ml/min. The analyte was eluted using a gradient of mobile phase A (0.1% formic acid in water) and mobile phase B (0.1% formic acid in methanol) as follows: 0-3.5 min: 6% B- 90% B; 3.5- 6.5 min: 90% B, 6.5-7 min, 90% B-6% B, 7-10 min, 6% B. The injection volume was 5 µL. The AB Sciex QTrap 3200 mass spectrometry was operated in positive electrospray ionization (ESI) mode using the following instrument settings: Curtain Gas: 10 ml/min; Collision Gas (CAD): medium; IonSpray Voltage (IS): 5500; Temperature: 650 °C; Ion Source Gas 1: 60; Ion Source Gas 2: 40; Declustering Potential (DP): 50; Entrance Potential (EP): 12; Collision Energy (CE): 42; Collision Cell Exit Potential (CXP): 6. PZ was quantified in MRM mode using the following mass transitions: 438.1/ 357.0. Data were acquired and processed using AB Sciex Analyst software (Version1.4.1).

Gene set enrichment analysis of transcription data

Gene set enrichment analysis was performed as reported previously[3] using the GSEA desktop application. GSEA was used to quantify enrichment of individual gene sets (e.g. OS up genes) or for multiple gene sets in transcription data for HLCs obtained from microarray. For this, unfiltered microarray expression data (i.e. all 20606 genes) was used and for each HLC, genes were rank ordered according to their fold change in PZ-treated compared to control samples. For the OS up gene set, enrichment was examined by determining where along the rank-ordered expression data, genes from this set fell. GSEA generates an enrichment score (ES) which is a measure of the degree to which a gene set is over-represented at the extremes of the entire ranked gene list. Significance of enrichment was assessed by using gene set permutation (1000 times) for random gene sets of similar size. The gene set permutation

is recommended when fewer than 7 samples are present for each phenotype, in this case, PZ or control, for which there were three samples each for each HLC. For single gene set GSEA the p-value of enrichment was the key output parameter considered and $p < 0.05$ was considered statistically significant. For investigating biological pathways affected by PZ, GSEA was used in combination with annotated gene sets –Hallmark (n=50), C2 KEGG pathways (n=186) C2 canonical pathways (n=1026 for gene sets with more than 5 genes) - available from the Molecular Signatures Database (MSigDB). Positive and negative values of NES indicated that pathways were upregulated and downregulated, respectively, in PZ-treated group compared to control group. To use GSEA to compare biological pathways distinctly affected in HT and NHT HLCs as a result of PZ treatment, expression data from PZ-treated HLCs was first normalized to their respective controls. Samples were labeled HT-PZ (including HT1, HT2 and HT3) or NHT-PZ (NHT1, NHT2) and genes were ranked by their fold change in HT-PZ compared to NHT-PZ. GSEA was done for Hallmark and KEGG pathways, or with the single gene set corresponding to iron-metabolism genes on this rank ordered dataset. Positive NES show upregulation and negative NES show downregulation of particular pathways in HT-PZ, with respect to NHT-PZ. When performing GSEA with multiple gene sets, p-value and false discovery rate-adjusted q value (FDR) was taken into account and only gene sets with p-value < 0.01 and FDR < 0.3 were considered statistically significant.

Verification of expression of genes in pazopanib-treated HLCs by qRT-PCR

For differentially expressed genes identified by SAM analysis and for selected Nrf2 genes, RNA from HLCs treated with 100 µM PZ or DMSO for 24 hours was used to make cDNA using the same procedure for cDNA synthesis described in “iPSC characterization”. Quantitative RT-PCR was done using primers listed in “Primers” section.

Primers

The following primer sequences or catalog numbers were used in the study for RT-PCR or qRT-PCR for the indicated genes:

Gene	Forward	Reverse
OCT4	GGGTTTTTGGGATTAAGTTCTTCA	GCCCCCACCCTTTGTGTT
SOX2	CAAAAATGGCCATGCAGGTT	AGTTGGGATCGAACAAAAGCTATT
NANOG	TTTGGAAGCTGCTGGGGAAG	GATGGGAGGAGGGGAGA
FLK-1	AAGGTGACAGGAAAAGACGAACT	TCCCCTCCATTGGCCCGCTTAAC
PAX-6	AACAGACACAGCCCTCACAAACA	CGGGAACTTGAACTGGAACTGAC
AFP	AGAACCTGTCACAAGCTGTG	GACAGCAAGCTGAGGATGTC
AKR1C1	TGTGCGATATTTGACCCTTGA	TGCTGTAGCTTGCTGAAATCAC
AKR1C2	CCTAAAAGTAAAGCTCTAGAGGCCGT	GAAAATGAATAAGATAGAGGTCAACATAG
ANKRD1	AGTAGAGGAACTGGTCACTGG	TGTTTCTCGCTTTTCCACTGTT
ASNS	GGAAGACAGCCCCGATTTACT	AGCACGAACTGTTGTAATGTCA
ASS1	AGGAAAGGGGAACGATCAGGT	GTGTTGCTTTGCGTACTCCA

NCOA7	AAAGACGCTCTACCGGAAATCG	CGCCTGTGCCATAATAGTGGT
SLC7A11	GCGTGGGCATGTCTCTGAC	GCTGGTAATGGACCAAAGACTTC
SOD2	TTTCAATAAGGAACGGGGACAC	GTGCTCCCACACATCAATCC
TGFB2	AAAGCCAGAGTGCCTGAACA	AGCGCTGGGTTGGAGATG
UBE2L6	TGGACGAGAACGGACAGATTT	GGCTCCCTGATATTCGGTCTATT
ABCC3	TGCTCTCCTTCATCAATCCA	TGGGGTTGGAGATAAACCTG
G6PD	TGAGCCAGATAGGCTGGAA	TAACGCAGGCGATGTTGTC
GCLC	CCCTCGCTTCAGTACCTTAAC	GACAGCAATTGCCCATTCCA
GPX2	GTGCTGATTGAGAATGTGGC	AGGATGCTCGTTCTGCCCA
GSR	ACCCCGATGTATCACGCAGTTA	TGTCAAAGTCTGCCTTCGTTGC
HMOX1	CTGCTCAACATCCAGCTCTTTG	AGTGTAAGGACCCATCGGAGA
ME1	CTGCTGACACGGAACCCTC	GATCTCCTGACTGTTGAAGGAAG
NQO1	CAAAGGACCCTTCCGGAGTAA	ACTTGGAAGCCACAGAAATGC
SOD1	GAAGGTGTGGGGAAGCATTA	ACATTGCCCAAGTCTCCAAC
TKT	TCATCGAGTGCTACATTGCTG	GCCATGCGAATCTGGTCAAAG
TXNRD1	ACGGTGATGCTGGCAATAGG	CTGGGGTGAGCTCCACCTTA
UGDH	CATCCAGGTGTTTCAGAGGATGAC	GAATGCGTTCATAATCCAATTCC
18S rRNA	AACTTTCGATGGTAGTCGCCG	CCTTGGATGTGGTAGCCGTTT
ACTB	CAAGATCATTGCTCCTCCTG	CCACATCTGCTGGAAGGTG
GAPDH	GTGGACCTGACCTGCCGTCT	GGAGGAGTGGGTGTCGCTGT
ALB	HQP005047 (Genecopoeia)	
AAT	HQP013122 (Genecopoeia)	
CK18	HQP054073 (Genecopoeia)	
ASGPR	HQP011276 (Genecopoeia)	
CYP1A1	HQP003772 (Genecopoeia)	
CYP1A2	HQP003774 (Genecopoeia)	
CYP3A4	HQP003836 (Genecopoeia)	
CYP3A7	HQP003801 (Genecopoeia)	
MRP2	HQP002260 (Genecopoeia)	
UGT1A1	HQP013615 (Genecopoeia)	
UGT1A3	HQP013616 (Genecopoeia)	

The following primer sequences or catalog numbers were used for determining genetic polymorphism by Sanger sequencing:

Gene	SNP	Forward	Reverse
HFE	rs2858996	GAGGTTGAAGATGATGGGAGGTC	TGTATGAGCTGGCTTCTTTCT
	rs707889	CTCAGTCAATCTGACCGTTTG	GGACCTTCCTTCAGGGACTTT
UGT1A1	rs4148323	GCAGCAGAGGGGACATGAA	GATCACACGCTGCAGGAAAG
	rs4124874	CTCACCAGAACAAACTTCTGAG	GATCCCTTGCTGTTCCCCAA
	rs8175347	GCTCCACCTTCTTTATCTCTGAA	GATCAACAGTATCTTCCCAGCA
CYP3A4	rs2740574	CACACTCCAGGCATAGGTAAAG	GGTTCTTATCAGAAACTCAAGT
CYP1A2	rs762551	AGGTATCAGCAGAAAGCCAGCAC	GCTGAGGGTTGAGATGGAGACAT
	rs12720461	AAGCTAGTGGGGACAGAAAGA	TTAAAAATGGCTTAGTCCAAACTG
CYP2C8	rs11572103	ACTCACAACAAAGTGCTTAA	CATTACTGGCCTGATCATTT
	rs10509681	CCTGCTGAGAAAGGCATGAA	CTACGTGATGTCCACTACTT
	rs1058930	GTTGCTCTTACACGAAGTTA	CAAGCATTACTGGCCTGATC
CYP3A5	rs776746	CGTATGTACCACCCAGCTT	GACACACAGCAAGAGTCTC

	rs28365083	GAGAGGACTTGCAAGAAAGATGT	CCCTGGAGACTTGTACCTT
ABCB1	rs3213619	CATCTGTGGTGAGGCTGATT	CGGCCACCAAGACGTGAAAT
	rs1128503	CCTAGTGAACAGTCAGTTCCTA	TGGACTGTTGTGCTCTTCC
	rs2032582	GGTTGGCAACTAACACTGTT	GAGCATAGTAAGCAGTAGGG
	rs1045642	CCCATCCTGTTTGACTGC	CATGCTCCCAGGCTGTTTAT
ABCG2	rs2231137	GTATTGTCACCTAGTGTTTGCA	CTTAACACAGCTCCTTCAGTA
	rs2231142	AACTGCAGGTTCATCATTAGC	CCTAACTCTTGAATGACCCTG
SLCO1B1	rs2306283	CTCAGGTGATGCTCTATTGAGT	GTACTCTGGTAATTTGGGGAA
	rs4149056	CCCAGTCTCAGGTATGTATTTA	TTTACTAGATGCCAAGAATGC
VEGFA	rs699947	CTGACTAGGTAAGCTCCCT	TGGCTATTTCCAGGCTGC
	rs833061	GTGAATGGAGCGAGCAG	TGAGAGCCGTTCCCTCT
	rs3025039	CGACAGAACAGTCCTTAATCC	GTGTCTACAGGAATCCCAGA
VEGFR2	rs2305948	CCTGACAAATGTGCTGTTCT	TCAGCATCAGCATAAGAAACT
	rs1870377	CTGGAAGTCCTCCACAC	TAGGCTGCGTTGGAAGTT
HIF1A	rs11549465	GACTTGGAGATGTTAGCTCC	GGCATTAGCAGTAGGTTCTTG
	rs11549467	CCAATGGATGATGACTTC	GGTGGCATTAGCAGTAGGTTC
GPX1	rs8179169	GCTGGCCTCCCCTTACAGT	GTAGTACCTTGCCCCGCA
	rs1050450	CACCGCGCTTATGACCGA	ACTGGGATCAACAGGACCAG
	rs1800668	TCAGGCCCGGAAAACTG	CGAGAAGGCATACACCGA
TXN2	rs2281082	CCTTGCTTACTGGCTACCTG	CGAGGTTAGAGAAGATGGTG
CAT	rs769214	GCACCTGAGGAGGTGTAG	CAGATGGGTGTTGATTTCCTC
PPARG	rs1801282	GACTCATGGGTGTATTCACAAA	CCTTACATAAATGCCCCCAC

SUPPLEMENTARY REFERENCES

1. **Tumilowicz, J.J. et al. Presence of retrovirus in the B95-8 Epstein-Barr virus-producing cell line from different sources. *In Vitro* 20, 486-492 (1984).**
2. **Rajesh, D. et al. Human lymphoblastoid B-cell lines reprogrammed to EBV-free induced pluripotent stem cells. *Blood* 118, 1797-1800 (2011).**
3. **Subramanian, A. et al. Gene set enrichment analysis: a knowledge-based approach for interpreting genome-wide expression profiles. *Proc Natl Acad Sci U S A* 102, 15545-15550 (2005).**
4. **Yu, J. et al. Human induced pluripotent stem cells free of vector and transgene sequences. *Science* 324, 797-801 (2009).**
5. **Leone, A.M. et al. Evaluation of felbamate and other antiepileptic drug toxicity potential based on hepatic protein covalent binding and gene expression. *Chem Res Toxicol* 20, 600-608 (2007).**

SUPPLEMENTARY DATA

Supplementary Table 1. Baseline clinical characteristics of five patients receiving pazopanib selected for iPSC generation.

**Patient**	**NHT1**[a]	**NHT2**[a]	**HT1**[b]	**HT2**[b]	**HT3**[b]
**Age, years**	43	53	65	71	51
**Sex**	F	M	M	M	M
**Tumor Stage (TNM)**	IV	IV	III	II	II
**Histology**	RCC	ccRCC	ccRCC	ccRCC	ccRCC
**Grade**	-	4	4	3	3
**ECOG PS**	-	1	0	1	0

[a] Patients without pazopanib-induced hepatotoxicity.

[b] Patients with on-treatment hepatotoxicity.

Abbreviations: ccRCC, clear-cell renal cell carcinoma; ECOG PS, performance status.

Supplementary Figure 1. Liver function tests of metastatic RCC (mRCC) patients receiving pazopanib treatment. (a-b) Liver function tests for mRCC patients NHT1 and NHT2, showing normal baseline and on-treatment serum levels of ALT, AST, ALP and bilirubin. **(c-e)** On-treatment elevation of ALT, AST levels (≥3x ULN) for patients HT1-3. PZ, pazopanib; ALT, Alanine transaminase; AST, Aspartate transaminase; ALP, Alkaline phosphatase; ULN, upper limit of normal. ULN defined as 66 IU/L for ALT, 42 IU/L for AST, 99 IU/L for ALP and 32 µmol/L for bilirubin.

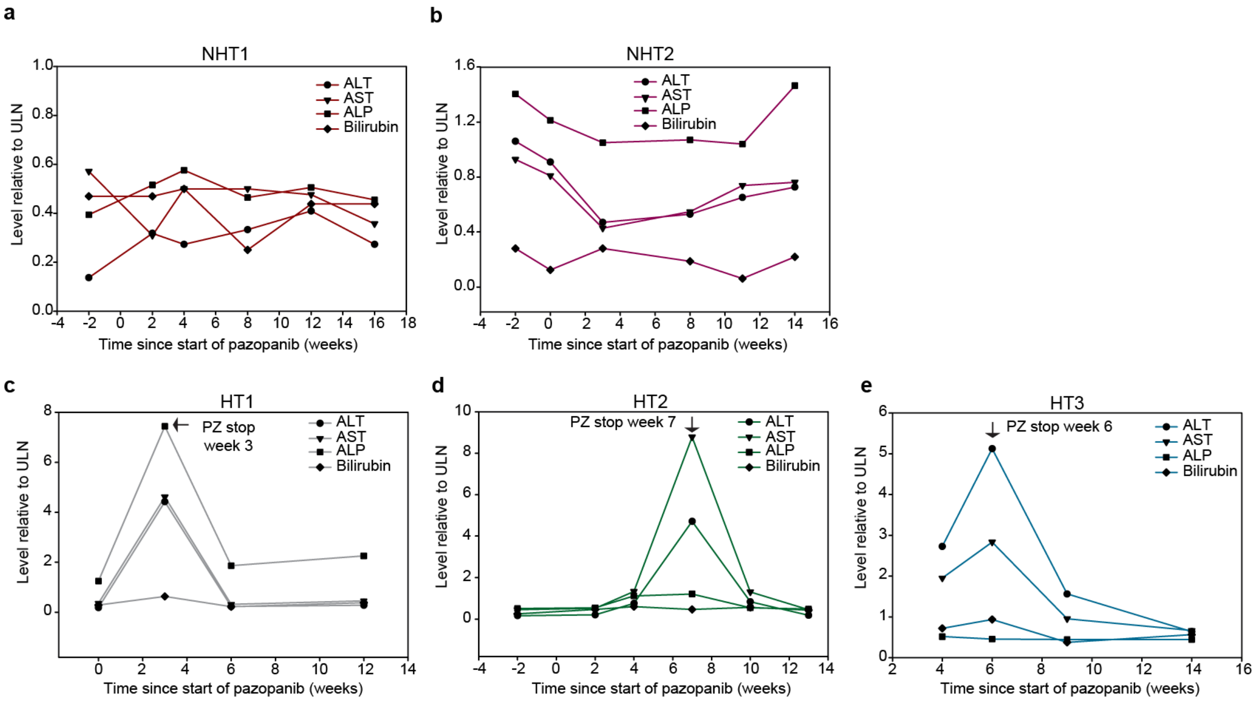

Supplementary Table 2. Genotyping of patient DNA for single nucleotide polymorphisms in *HFE* and *UGT1A1* genes. Sequencing of DNA for patients for specific polymorphisms in *HFE* and *UGT1A1* reported to be correlated with levels of elevated alanine transaminase and bilirubin, respectively, in patients receiving pazopanib. NHT1 and NHT2 are patients that did not manifest clinical hepatotoxicity, and HT1-3 are patients that had clinical hepatotoxicity to pazopanib.

			Case				
Gene	Polymorphism	rs number	NHT1	NHT2	HT1	HT2	HT3
HFE	1007-388G/A/T	rs2858996[a]	GG	GT	GG	GG	GG
	*1477G>A	rs707889	GG	GA	GG	GG	GG
UGT1A1	862-10021T>G	rs4124874	TG	TG	TG	TT	TG
	211G/A	rs4148323	GG	GG	GG	GG	AA
	TA6/TA7	rs8175347[b]	TA7/TA7	TA7/TA7	TA7/TA7	TA7/TA7	TA7/TA7

[a] Risk-associated genotype described for *HFE* rs2858996 is TT.

[b] Risk-associated genotype for *UGT1A1* rs8175347 is TA7/TA7.

Supplementary Figure 2. Reprogramming of patient EBV-immortalized lymphocytes to induced pluripotent stem cells. (a) The combination of two episomal plasmid encoding *OCT4*, *SOX2*, *SV40/*LT and *KLF4* (Plasmid 1) and *OCT4*, *SOX2*, *NANOG* and *LIN28* (Plasmid 2) was used to reprogram EBVi-lymphocytes (EBVi) to iPSCs. The plasmids were originally described by Yu *et al* [4] and can reprogram human somatic cells to iPSCs without genomic integration of reprogramming factors. **(b)** Schematic of the reprogramming process. Following single nucleofection of EBVi cells with plasmids in **a.**, colonies that emerged were transitioned from reprogramming medium to stem cell medium mTESR1. Time in days is indicated below. **(c)** Morphology of typical reprogrammed colony that appeared 33-53 days after start of reprogramming process (magnification, X40). **(d)** Live staining of TRA-1-60 in picked colonies (as in **c.**) confirming reprogrammed state (magnification, X40).

a

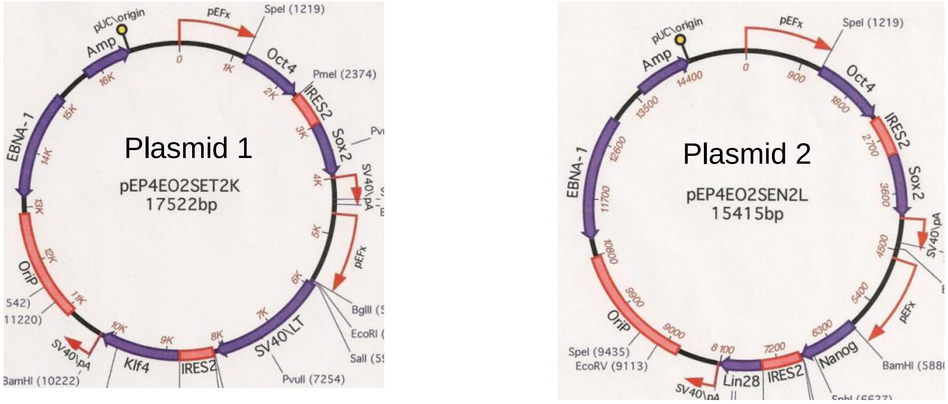

b

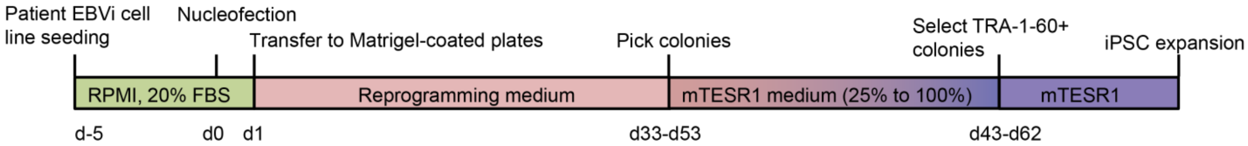

c

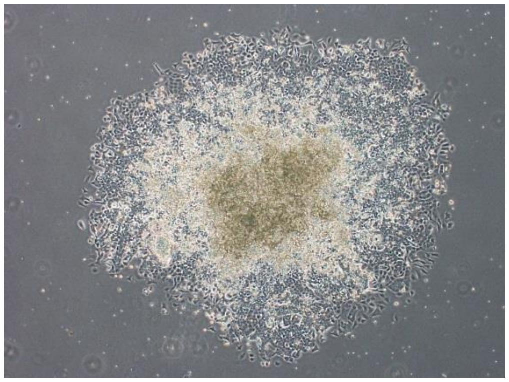

d

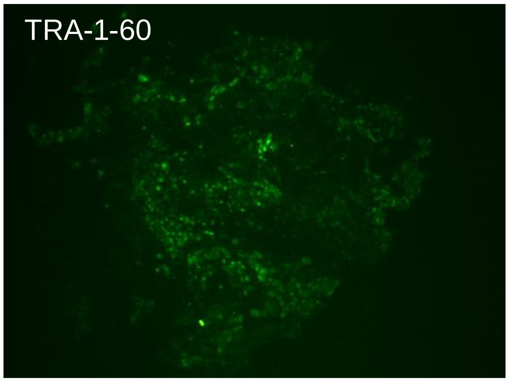

Supplementary Figure 3. Characterization of patient-specific iPSC lines. (a) Immunofluorescence analysis of pluripotency markers in iPSCs generated from EBVi cells of five patients. All five iPSC lines express transcription factors OCT4 and NANOG and surface antigens TRA-1-60 and SSEA4. Scale bar = 50 µm. **(b)** RT-PCR analysis of expression of pluripotency markers in EBVi and derived iPSC lines. Marker expression is present in iPSC lines and absent in EVBi cells. Human embryonic stem cells, H9, were used as a positive control for expression. NTC = no-template control.**(c)** RT-PCR analysis of expression of markers of endoderm (*AFP*), ectoderm (*PAX6*) and mesoderm (*FLK-1*) lineages in day 7 embryoid bodies (EB) generated from iPSC lines. Increased expression of each lineage marker is observed in EBs compared to the iPSC lines from which they were derived. Relative expression in EBs is indicated for each marker and is derived from intensity analysis of bands in image, done using Image J. NTC = no-template control. **(d)** Hematoxylin and eosin staining of teratomas derived from immunodeficient mice injected with HT2-iPSCs showing tissues representing all 3 germ layers, mesoderm (cartilage and SM, smooth muscle: arrow), ectoderm (NR, neural rosettes and PE, pigmented epithelium) and endoderm (RGE, respiratory glandular epithelium and GE, glandular epithelium). Scale bar = 200 µm. **(e)** Karyotype analysis showing normal karyotype for all iPSC lines, except HT1-iPSC (monosomy 21).

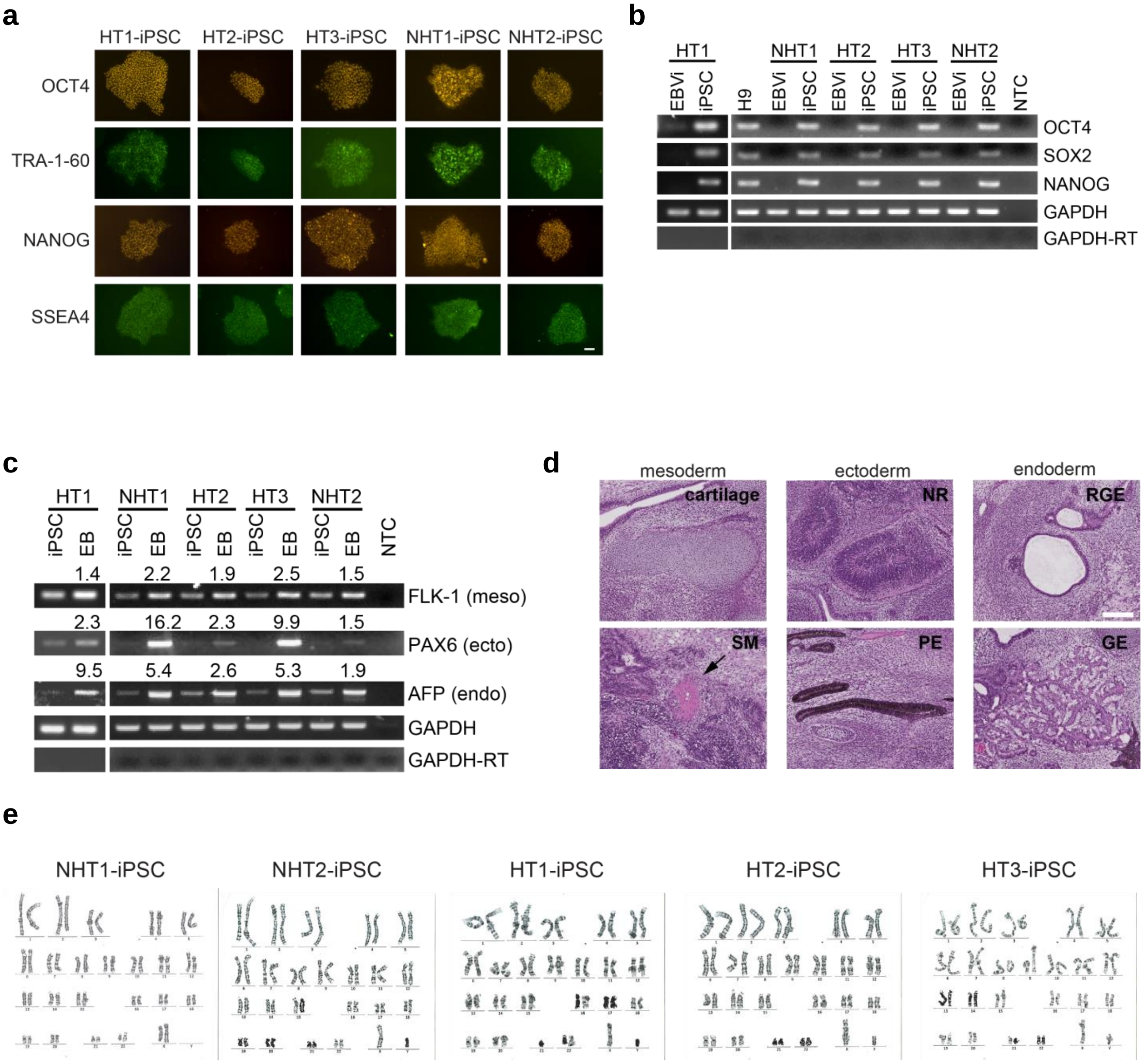

Supplementary Figure 4. Flow cytometry analysis of albumin+ cells in (a) HT and NHT iPSC-derived HLCs after 20 days of differentiation. (b) and undifferentiated HT and NHT iPSCs.

a

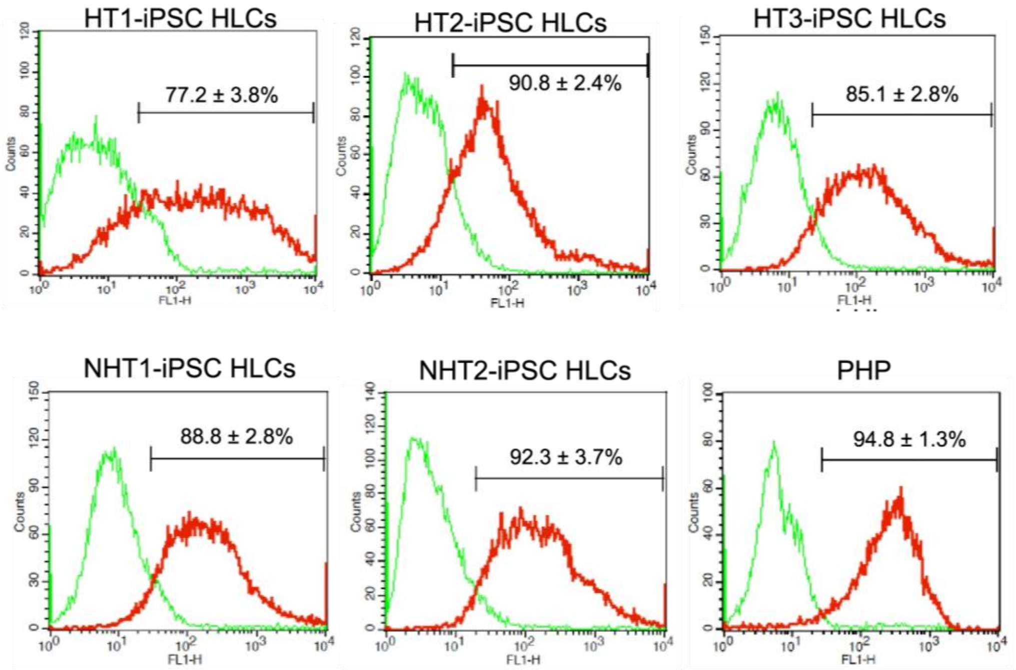

b

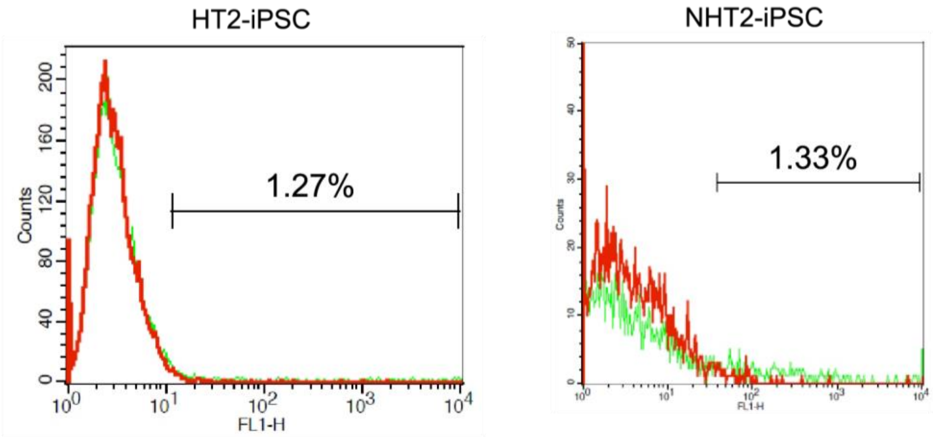

Supplementary Figure 5. Cell viability of undifferentiated iPSC lines after 24 hours incubation with different concentrations of pazopanib. Single iPSCs were plated and incubated with 10 uM ROCKi overnight to allow for cell attachment before being treated with PZ for 24 hours. Cell viability was measured using MTS assay. **(a-b)** iPSCs from non-hepatotoxic patients, NHT1 and NHT2. **(c-e)** iPSCs from hepatotoxic patients HT1, HT2 and HT3. Cell viability data are average ± S.D of duplicate samples.

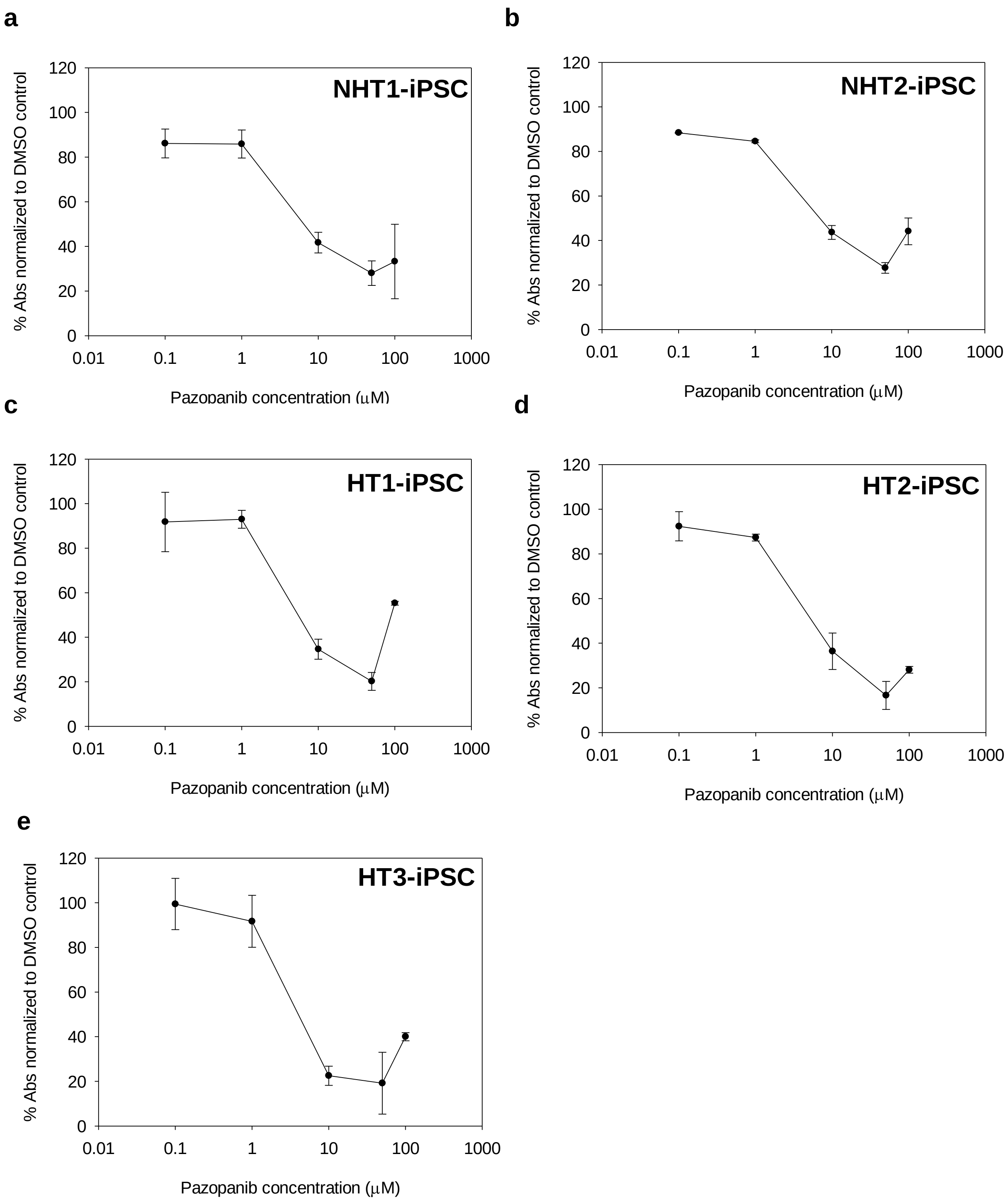

Supplementary Figure 6. Cell viability of undifferentiated iPSC lines after 24 hours incubation with different concentrations of APAP. Single iPSCs were plated and incubated with 10 uM ROCKi overnight to allow for cell attachment before being treated with PZ for 24 hours. Cell viability was measured using MTS assay. **(a-b)** iPSCs from non-hepatotoxic patients, NHT1 and NHT2. **(c-e)** iPSCs from hepatotoxic patients HT1, HT2 and HT3. Cell viability data are average ± S.D of duplicate samples.

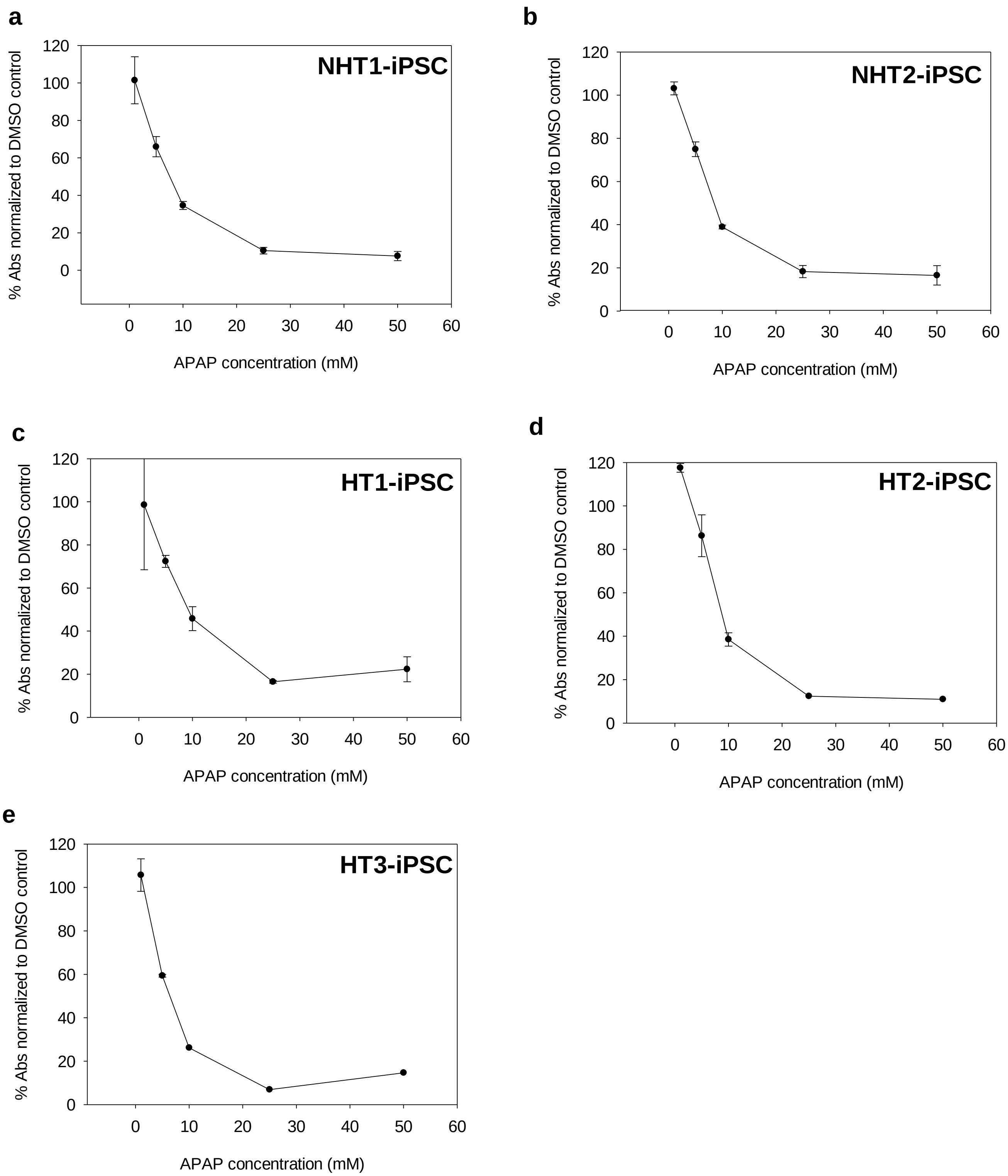

Supplementary Figure 7. Cell viability of patient-specific HLCs measured by ATP assay after 24 hours incubation with different concentrations of pazopanib. (a-b) HLCs derived from non-hepatotoxic patient iPSC lines, NHT1 and NHT2. **(c-e)** HLCs derived from hepatotoxic patient iPSC lines, HT1, HT2 and HT3. Data are means ± s.e.m. of 3 independent differentiation experiments.

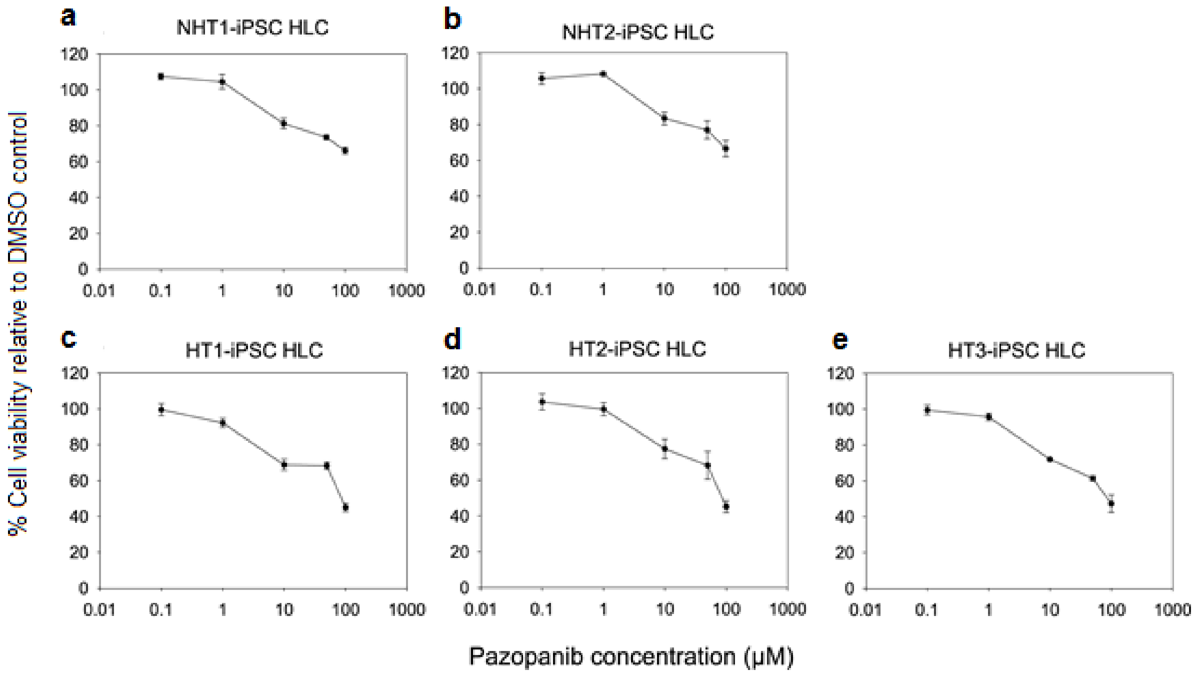

Supplementary Table 3. Gene expression changes in HLCs after PZ treatment. Top 50 differentially expressed genes in each HLC after exposure to 100 μM PZ for 24 hours as measured by microarray. Differential expression analysis was done using significance analysis of microarrays (SAM). Fold-change is with respect to respective vehicle control of each HLC and HLCs from hepatotoxic patients are HT1, HT2 and HT3.

NHT1		NHT2		HT1		HT2		HT3	
Gene	Fold change	Gene	Fold change	Gene	Fold change	Gene	Fold change	Gene	Fold change
UBE2L6	3.01	NPPB	0.10	AKR1C1	4.20	CLDN11	10.36	ASNS	5.47
GINS1	0.43	ASS1	7.33	GDF15	3.08	OXTR	0.07	IFIT3	4.20
MCM4	0.44	C4orf26	0.19	TM4SF1	2.46	IL11	0.12	AKR1C1	3.03
CDK1	0.43	PHGDH	3.38	AKR1C2	3.75	VTCN1	7.70	AKR1C2	2.83
TOP2A	0.43	CDKN1C	3.35	CXCL1	5.82	GREM1	0.14	CXCL1	2.15
KIF11	0.47	CCL2	0.18	AKR1C1	3.83	ASS1	7.13	SQSTM1	2.03
CDK1	0.43	TIMP3	0.34	KANK4	0.38	GREM1	0.15	AKR1C2	2.80
DBF4	0.53	SLC7A11	4.20	SRXN1	2.04	CTGF	0.11	SOD2	2.27
UHRF1	0.42	TGFB2	0.23	AKR1C2	4.17	CXCL14	10.24	GREM1	2.29
TTK	0.45	HGF	4.89	ATF3	3.17	NPPB	0.09	PSAT1	2.49
DLGAP5	0.45	CFLAR	0.28	GPRC5A	2.30	ACTG2	0.18	TPM4	0.46
RRM2	0.46	TIMP3	0.30	OLR1	2.06	GLIPR1	0.25	TM4SF1	1.84
UBE2C	0.48	INHBA	0.17	TM4SF1	3.02	TMEM158	0.12	RAB27B	2.03
TMEM97	0.54	SLC7A11	4.11	PTGDS	2.74	HEY1	4.80	SLC7A11	2.38
ASPM	0.47	PTX3	0.10	TM4SF1	2.64	GLIPR1	0.23	AKR1C1	2.39
ANLN	0.52	ASNS	4.49	HMOX1	2.47	CXCL14	10.33	CCDC80	0.41
PBK	0.50	TMEM158	0.19	CDK1	0.44	GLIPR1	0.27	RBM25	0.58
AREG	2.10	CDKN1C	4.33	CDK1	0.47	PIR	4.05	TUBB2A	1.88
CACYBP	0.58	GLIPR1	0.34	CDCA3	0.43	C4orf26	0.18	SOD2	2.29
CKS1B	0.52	IER3	0.35	PTGDS	2.99	INHBA	0.20	UBD	2.47
BUB1	0.51	CNN1	0.28	PTX3	2.30	METTL7A	3.46	TM4SF1	1.78
CDK1	0.48	CXCL14	2.73	CDK1	0.46	CNN1	0.13	PSAT1	2.18
GDF15	2.60	CTPS1	0.29	TACSTD2	1.94	CRISPLD2	2.83	SPP1	3.70
TOP2A	0.49	PCK2	4.30	CENPA	0.51	CDKN1C	4.74	GDF15	3.00
ROR1	0.53	APCDD1	3.55	HMGCS1	0.48	SLC7A11	3.70	CALD1	0.57
PRR11	0.53	SERPINE1	0.17	NUSAP1	0.50	RSPO2	5.47	CDC42BPA	0.45
NUSAP1	0.52	RBM24	0.19	DPP4	1.73	CDKN1C	3.86	SOD2	1.92
RGS5	0.50	CDKN1C	4.48	FANCI	0.57	SEMA6D	4.56	TOP1	0.53
KPNA2	0.64	CDH6	0.26	TOP2A	0.41	DDAH1	0.33	F2RL1	1.72
MCM3	0.56	GABRP	2.97	KRT7	1.96	CYR61	0.33	SERPINE1	1.76
AKR1C2	2.32	TIMP3	0.31	USP1	0.62	ANKRD1	0.05	MAFF	2.00
ANLN	0.47	UBE2L6	2.81	KIF11	0.48	XYLT1	0.20	MDM2	1.80
AKR1C2	2.37	OXTR	0.19	C1S	1.88	TGFB2	0.16	TUBB2A	1.92
AKR1C1	2.33	MT1E	0.25	LMNB1	0.52	PHGDH	3.84	CD9	1.77
EGR1	0.48	HBEGF	0.26	MELK	0.58	HBEGF	0.21	SLC7A11	2.30
CCNA2	0.50	MICAL2	0.42	CKS1B	0.54	SLC40A1	3.69	PDLIM3	2.02
SRSF1	0.60	MT1H	0.29	LBH	1.76	CDKN1C	4.05	CDKN1A	1.92
LMNB1	0.47	MT2A	0.26	SQSTM1	1.94	TXNIP	4.15	GPRC5A	1.85
TMPO	0.46	CDKN1C	3.82	TTK	0.55	AMD1	0.32	STX3	1.77
MCM7	0.49	SHMT2	2.27	TSC22D3	1.72	AKR1C1	2.84	AQP1	0.58
AKR1C1	2.79	HCFC1R1	2.70	AREG	2.33	TAGLN	0.31	PAPPA	1.69
CENPA	0.49	COL15A1	3.41	KRT6A	2.00	PDLIM3	0.25	NAMPT	1.67
SPDL1	0.62	GLIPR1	0.34	DLGAP5	0.52	ASNS	3.47	TOP2A	0.44
USP1	0.59	ACTC1	0.32	FEN1	0.58	CDKN1C	4.53	PTGES	1.60
CENPU	0.56	ANKRD1	0.11	UBE2C	0.44	CXCL1	0.18	HIST1H4C	0.54
HLA-E	1.54	RHOU	2.84	RRM2	0.48	ALDH6A1	3.30	MOSPD1	1.56
NEAT1	1.98	DUSP1	0.28	ANLN	0.56	MICAL2	0.35	SERPINE1	1.75
MAD2L1	0.54	C5orf46	0.24	DCN	1.96	GAS5	2.97	TAGLN	0.59
BUB1B	0.49	CDC42EP3	0.46	CCNA2	0.56	PTX3	0.30	CDH10	1.74
EPSTI1	2.36	IFITM1	2.52	KITLG	1.57	RBM24	0.24	KCTD12	0.66

Supplementary Figure 8. Overlap of transcriptional changes induced by PZ in HT- and NHT-HLCs
(a) Venn diagram depiction of differential gene regulation in HT-HLCs treated with PZ and list of genes present in their intersections. **(b)** Venn diagram depiction of gene regulation in NHT-HLCs treated with PZ and their overlap. Genes in bold are down-regulated. Numbers of upregulated (↑) and downregulated (↓) genes are indicated with the Venn diagram.

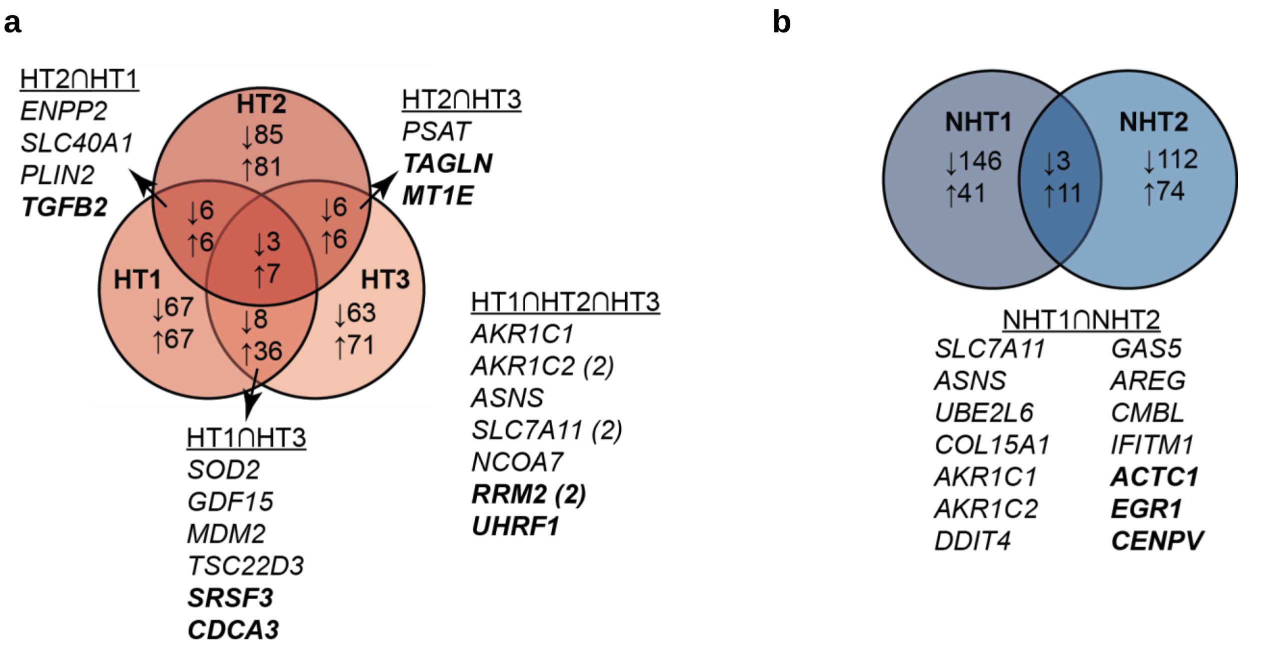

Supplementary Figure 9. Validation of gene expression changes induced by PZ. Expression verification by qRT-PCR of genes that were identified to be differentially expressed across HLC lines using SAM analysis of microarray data. PZ treatment (100 µM) was done for 24 hours. For each HLC, fold-change represents the expression in PZ-treated samples relative to their vehicle control, DMSO. Data are presented as mean ± SD from triplicate measurements.

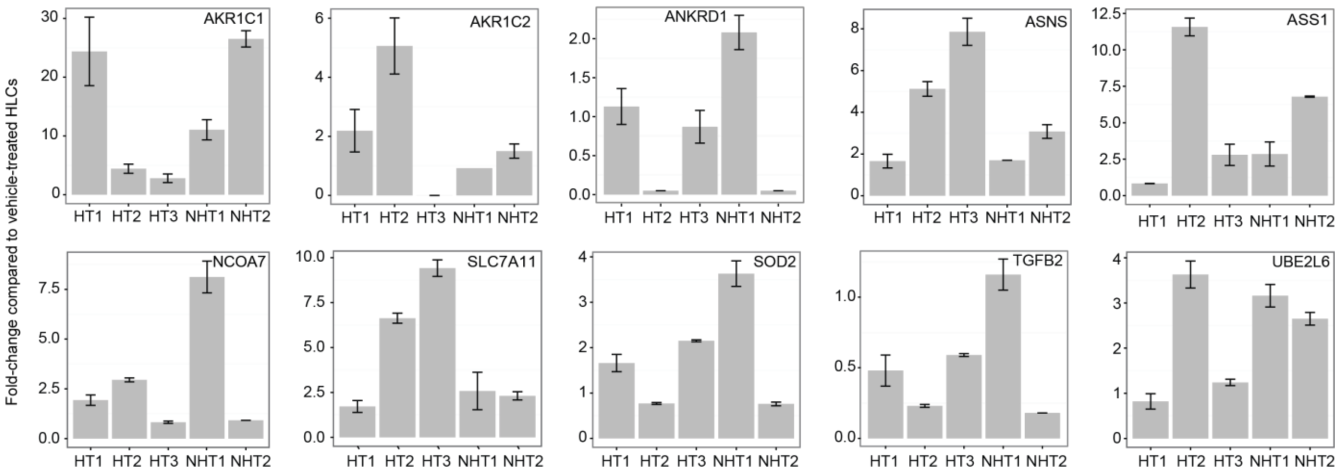

Supplementary Table 4. Enriched of functional gene sets in PZ-treated HLCs. Gene set enrichment analysis (GSEA) was done for expression data collected after exposure of HLCs to 100 µM PZ for Hallmark gene sets from MSigDB (n = 50) were analyzed. The table shows gene sets that are either uniformly induced or uniformly repressed in HLCs, i.e. constituent genes' expressions are coordinately upregulated or downregulated, respectively, as a result of treatment with PZ. Gene sets labeled as variable are significantly enriched but their enrichment is not in the same direction for all HLCs. ,

		NHT1			NHT2			HT1			HT2			HT3		
		NES	p	FDR	NES	p	FDR	NES	p	FDR	NES	p	FDR	NES	p	FDR
induced	Interferon alpha response	2.87	0	0	1.86	0	0.	2.40	0	0	2.08	0	0	2.41	0	0
	Interferon gamma response	2.70	0	0	1.31	0.04	0.25	2.27	0	0	1.19	0.12	0.26	2.18	0	0
	Bile acid metabolism	1.39	0.03	0.07	1.49	0.01	0.09	1.50	0.01	0.02	1.52	0.01	0.07	0.67	1.00	1.00
variable	IL6 JAK-STAT signaling	2.14	0	0	1.03	0.40	0.57	2.10	0	0	-0.94	0.61	0.63	2.09	0	0
	TNFα signaling	1.82	0	0	-2.90	0	0	2.33	0	0	-2.99	0	0	2.63	0	0
repressed	MYC targets	-2.87	0	0	-2.12	0	0	-2.08	0	0	-1.54	0	0.02	-1.74	0	0
	E2F targets	-3.67	0	0	-2.30	0	0	-3.15	0	0	-2.44	0	0	-2.72	0	0
	TGFβ signaling	-1.61	0.01	0.01	-1.75	0	0	-0.97	0.50	0.57	-1.59	0.01	0.02	-0.90	0.67	0.95

Abbreviations : NES, normalized enrichment score; FDR, false discovery rate

Supplementary Figure 10. Expression verification by qRT-PCR of genes regulated by Nrf2 in HLCs. Selected Nrf2 target genes were quantified by qRT-PCR in HLCs treated with 100 µM PZ for 24 hours. For each HLC, fold-change represents the expression in PZ-treated samples relative to their vehicle control, DMSO. Data are presented as mean ± SD from triplicate measurements. The dark grey bars correspond to HT1, HT2 and HT3.

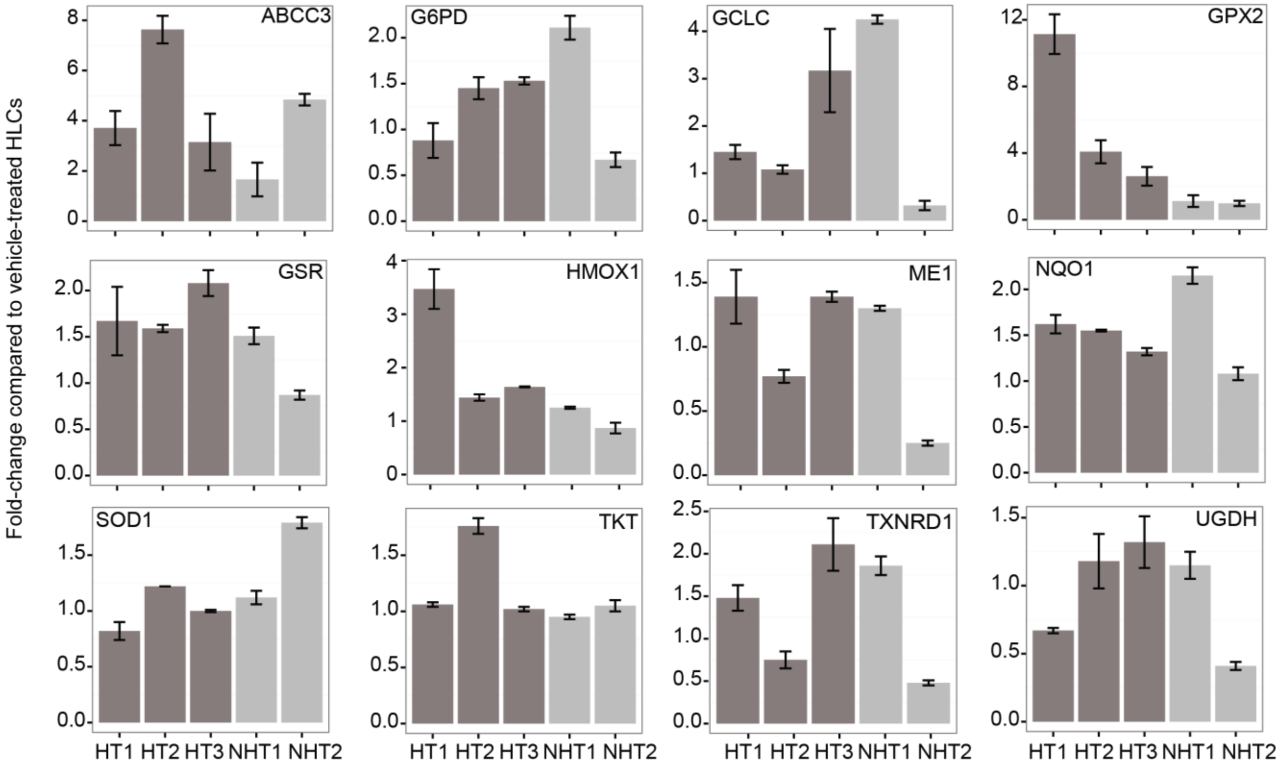

Supplementary Table 5. List of genes corresponding to the drug-induced hepatocyte-specific oxidative stress signature. The list is based on a consensus signature of drug-induced oxidative stress in the rat liver identified by Leone *et al*[5]. Transcripts of the listed genes are induced upon drug-induced oxidative stress and are referred to as OS ‘up’ signature genes.

OS ‘up’ signature genes
AKR7A2
ALDH1A1
EPHX1
NQO1
HSP90
UGDH
PGRMC1
ST13
GSTA1
ME1
TXNRD1
HMOX1
TKT
ENPEP
ABCC3
GSTM2

Supplementary Table 6. Genes differing significantly in expression between HT- and NHT-HLCs as a result of PZ exposure are potentially unique to idiosyncratic hepatotoxicity in HT-HLCs. Expression from PZ-treated HLCs was normalized to their respective controls and samples grouped as HT-HLCs (HT1, HT2 and HT3) or NHT-HLCs (NHT1 and NHT2). Differential expression between HT and NHT samples was assessed by SAM to identify the most extreme of expression changes that occur in hepatotoxic lines (HT) compared to non-hepatotoxic lines (NHT). Fold change represents HT vs NHT comparison.

Gene	Fold change	Gene	Fold change
HPGD	1.69	CDC123	1.20
KCNE4	1.44	DHX9	1.37
SPINK1	1.45	GLS	1.26
FLT1	1.77	ABLIM1	1.45
CIRH1A	1.40	DNAJB6	1.33
UPP1	1.35	CLIP4	0.73
COL6A1	0.69	GPRC5A	1.62
ERRFI1	1.25	NIFK	1.26
TFRC	1.53	MRPS18C	1.21
SOCS2	1.33	CCNT1	1.21
HNRNPF	1.31	DCAF13	1.22
MRPS30	1.28	HSPA8	1.28
LIPG	0.65	DDX47	1.20
COL1A2	0.75	NUAK1	1.36
COL1A1	0.71	PSMC4	1.23
RNF138	1.32	JAG1	1.34
TPD52L1	0.78	HSPA4	1.30
MGP	0.53	IDH3B	1.22
HSPA9	1.27	STXBP6	1.29
SULF1	1.39	EIF1AX	1.19
NOP56	1.29	LAMA5	0.77
DNAJA2	1.24	DDX18	1.22
RSL1D1	1.26	CDKN2B	1.98
S1PR3	0.74	STXBP6	1.32
NOL11	1.26	CCT6A	1.27
PPID	1.26	HSP90AB1	1.32
MFAP5	1.59	EIF5	1.20
RB1CC1	1.26	ZNF770	1.19
WBSCR22	1.35	MRPL30	1.19
MAP1B	0.75	TIMM50	1.20
CCT6A	1.24	WNT5A	1.26
DLC1	1.43	SULF1	1.30
GNPDA1	1.25	LAPTM4B	1.34
ADAMTS15	0.45	UBE2D2	1.17
WLS	1.27	CEBPB	0.80
RPF2	1.24	TFAM	1.29
DRG1	1.21	CCT8	1.17
GRSF1	1.25	MRPS23	1.22
RGS5	1.54	ATG101	1.32
MATR3	1.35	PKIA	0.68
GTF2E1	1.27	TMEM245	0.82
DIMT1	1.22	BFAR	1.19
VWA5A	1.23	CSNK1A1	1.23
EIF1AX	1.34	RAE1	1.20
FAM60A	1.24	CCT3	1.18
CUX1	0.75	TCEAL3	0.79
HPGD	1.53	STIP1	1.28
EIF5	1.22	ACOT7	1.24
DIMT1	1.20	CENPV	1.30
UTP18	1.29	MFAP5	1.61

Supplementary Figure 11. Expression of genes involved in iron metabolism in HLCs. Differential modulation of genes involved in iron metabolism in HT-HLCs compared to NHT-HLCs treated with PZ, particularly *TFRC* and *HFE*. Gene functions in iron metabolism are noted. Genes denoted in blue are pharmacological targets of PZ, that have overlapping regulatory elements with iron metabolism genes. The heatmap shows log2 fold-changes (log FC) compared to control.

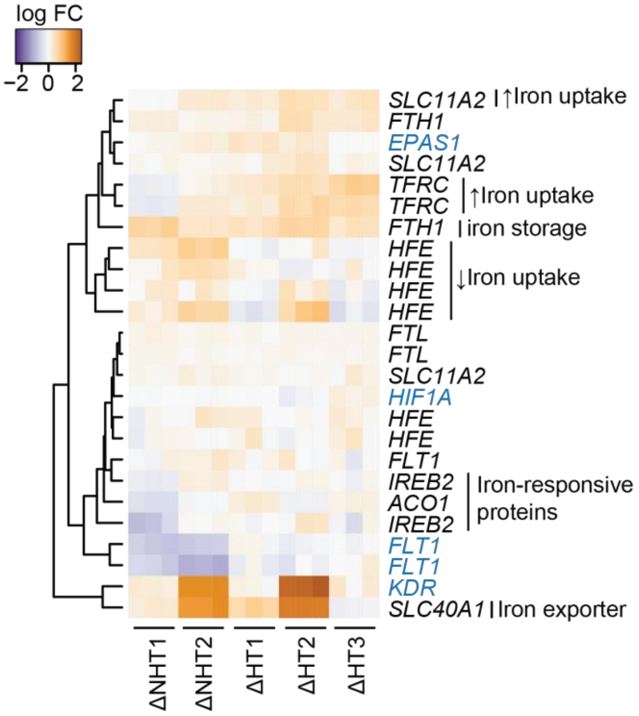

Supplementary Figure 12. Enrichment plots for gene sets from Hallmark and KEGG pathways comparing HT and NHT-HLCs treated with PZ. GSEA showed significant enrichment for **(a)** Oxidative phosphorylation and **(b)** Spliceosome in HT-HLCs. Enrichment was seen for **(c)** Metabolism of xenobiotics and **(d)** Linoleic acid metabolism in NHT-HLCs. The ES, normalized enrichment score (NES), and false discovery rate-adjusted q value (FDR) are indicated for each gene set.

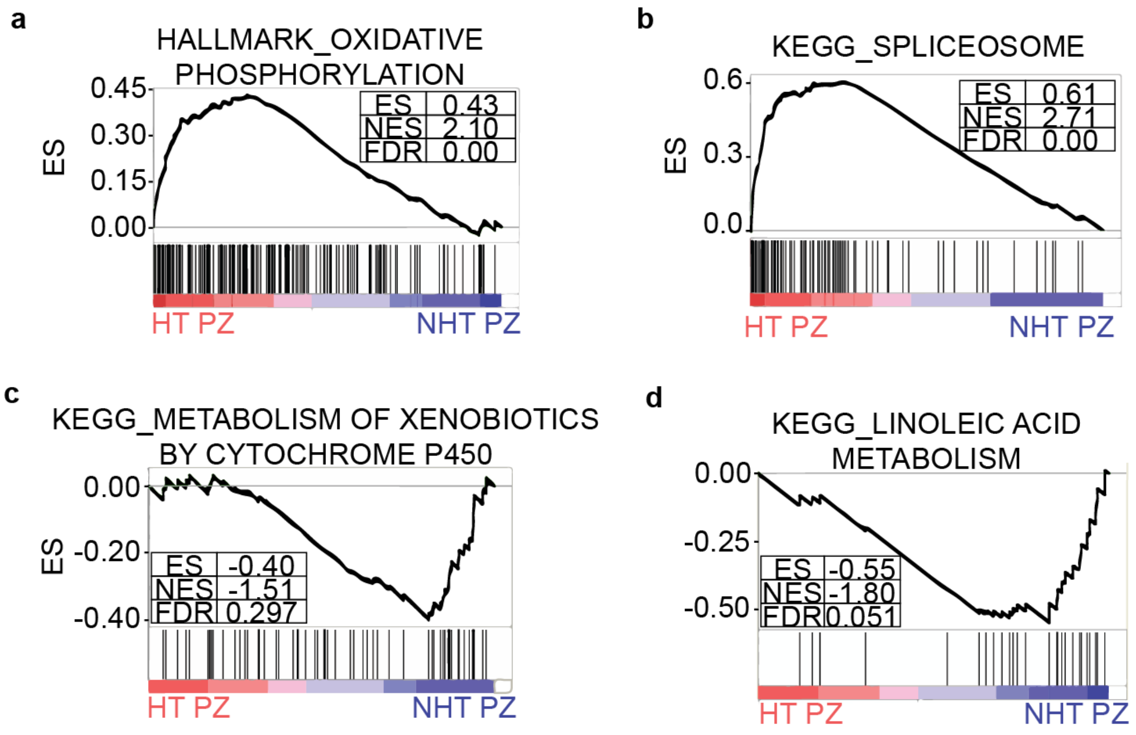

Supplementary Table 7. Gene sets related to RNA processing are highly enriched in HT-HLCs treated with PZ. The table shows output from gene set enrichment analysis (GSEA) on expression data of PZ-treated HT-HLCs (HT-PZ) and NHT-HLCs (NHT-PZ) using MSigDB curated canonical pathway gene sets (n = 1026). In comparing HT-PZ to NHT-PZ , gene sets related to RNA processing were enriched in HT-PZ group and were the topmost enriched gene sets in this analysis.

Rank	Gene set	SIZE	ES	NES	p-val	FDR
1	KEGG_SPLICEOSOME	96	0.61	2.71	0	0
2	KEGG_RNA_POLYMERASE	28	0.69	2.34	0	0
3	KEGG_BASAL_TRANSCRIPTION_FACTORS	33	0.61	2.19	0	0
4	KEGG_RNA_DEGRADATION	51	0.54	2.06	0	0
5	KEGG_RIBOSOME	79	0.4	1.71	0.002	0.069

Abbreviations : ES, enrichment score; NES, normalized enrichment score; FDR, false discovery rate

Supplementary Figure 13. N-acetyl cysteine can partially rescue the viability of HT-HLCs. HLCs were exposed to 10 µM PZ with co-incubation of N-acetyl cysteine (NAC) and viability was measured 24 hours later. Data are presented as mean ± s.e.m from two independent experiments. *p < 0.05.

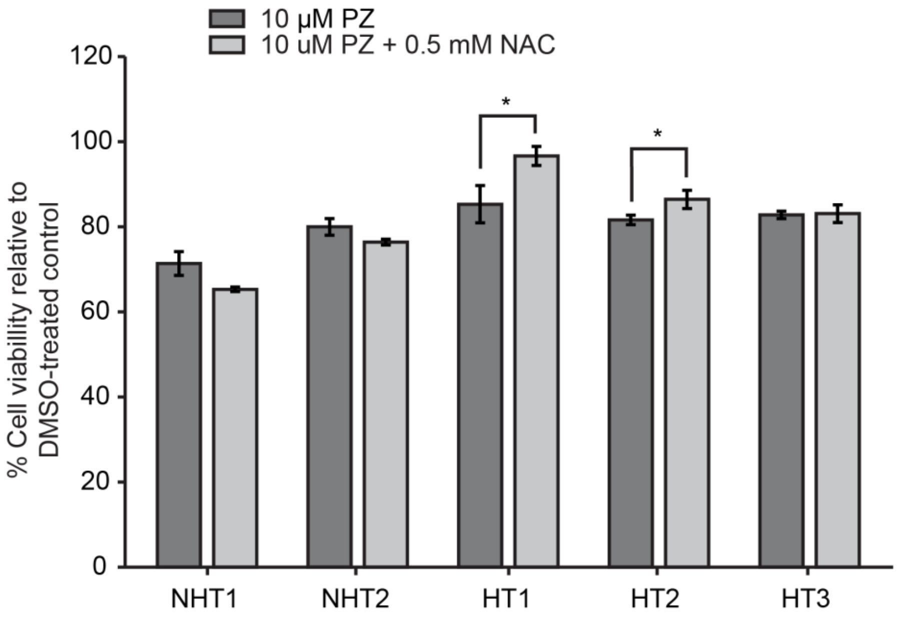

Supplementary Figure 14. Detection and quantification of reactive oxygen species (ROS) accumulation in PZ-treated HLCs. HLCs were treated with 10 µM PZ or 50 mM APAP 4 hours and in the final hour 10 µM CellROX Green was added to the medium. Fluorescence intensity of CellROX was measured by flow cytometry. Compared to vehicle-treated control HLCs, a rightward shift in the profile is noted and indicates the increased intensity of CellROX fluorescence indicating accumulation of ROS. The top panel is for 10 µM PZ, the middle panel for 50 mM APAP and the bottom panel is for 10 µM menadione (K3) as positive control for generation of ROS. The mean fluorescence intensity was quantified and compared to control samples. Representative traces from two independent experiments are shown.

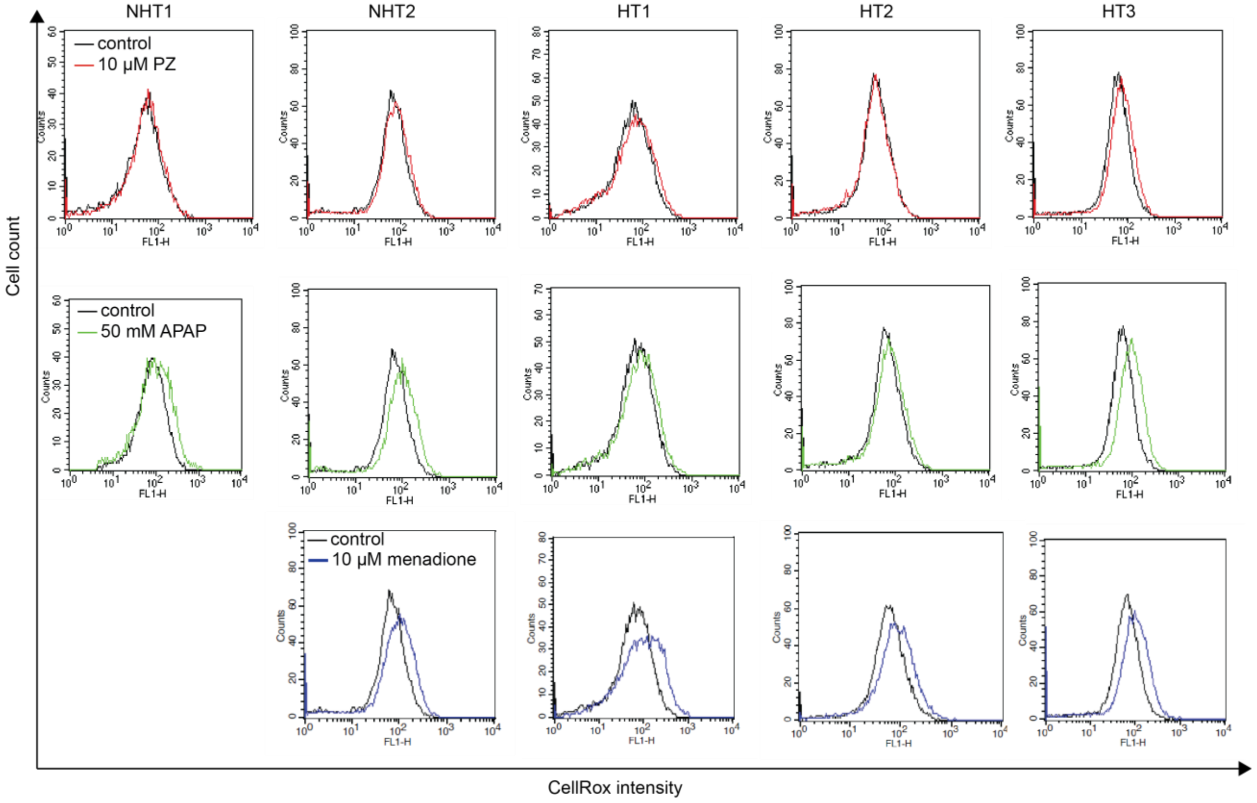

Supplementary Figure 15. Glutathione depletion and ROS accumulation in HLCs induced by acetaminophen (APAP). (a) Glutathione depletion is correlated to ROS accumulation. Greater depletion of GSH, indicated by a lower GSH/GSSG is accompanied by lesser ROS accumulation in HLCs. **(b-c)** Correlation between GSH depletion in APAP-treated HLCs and basal activity of CYPs in HLCs. Relative GSH/GSSG ratio is inversely correlated with basal CYP1A2 activity levels **(b)** but not with CYP3A4 activity levels **(c)**. Each data point represents an individual HLC, indicated by same color legend in **a**. Correlation coefficient, R and p-values are determined by Pearson's correlation.

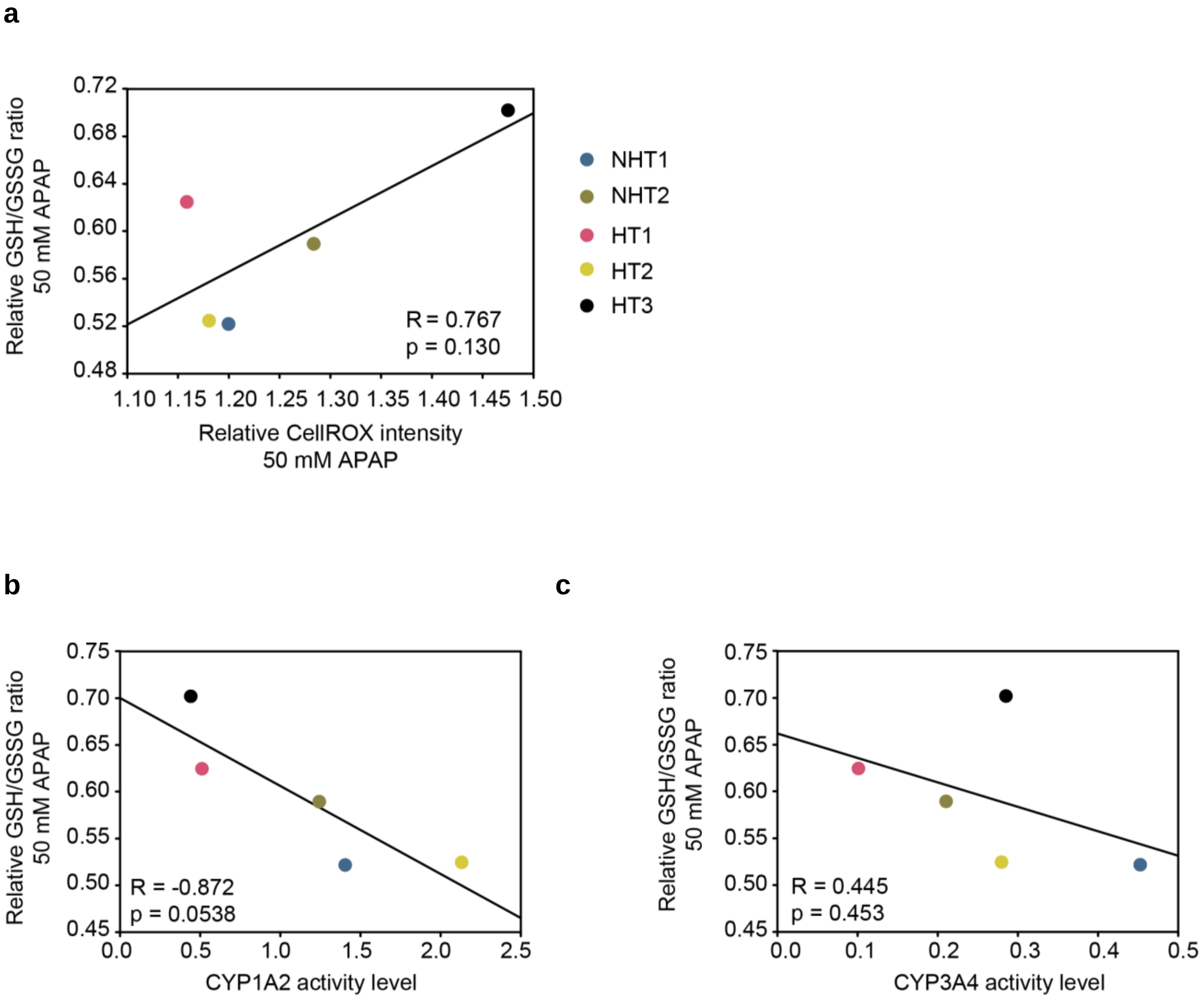

Supplementary Figure 16. Quantification of intracellular PZ in HLCs by Liquid Chromatography-Mass Spectrometry (LC-MS). Intracellular accumulation of parental PZ was meaured in samples prepared from HLCs treated with 100 µM PZ for four hours. The extent of intracellular PZ accumulation is comparable in all HLCs (ANOVA, $p = 0.961$).

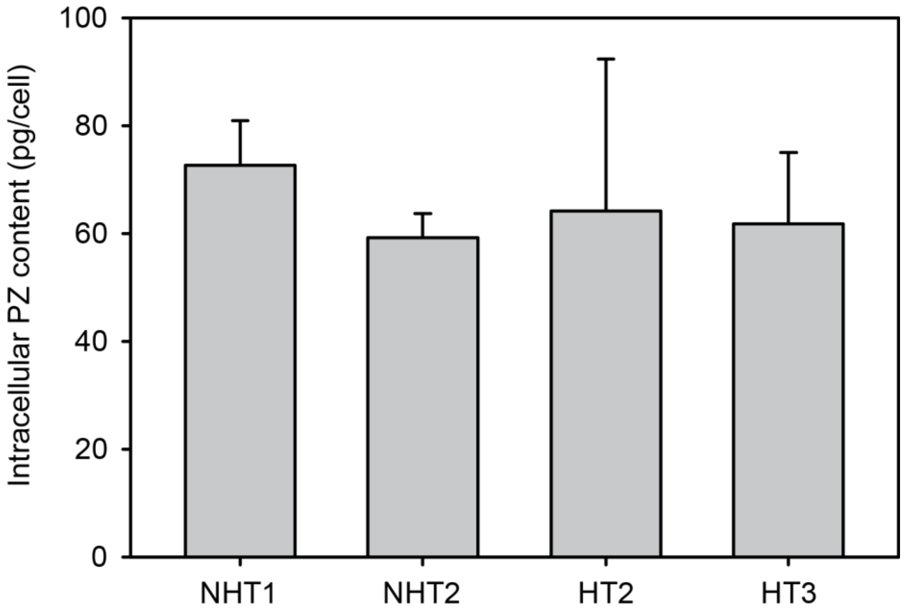

Supplementary Table 8. Genotyping of patient DNA for single nucleotide polymorphisms (SNPs) in CYPs involved in metabolism of pazopanib

		Case				
Gene	SNP	NHT1	NHT2	HT1	HT2	HT3
CYP3A4	-392A/G (rs2740574)	AG	AG	GG	GG	GG
CYP1A2	-163C/A (rs762551)	AA	AA	CC	AC	CC
	-729C/T (rs12720461)	CC	CC	CC	CC	CC
CYP2C8	805A/T (rs11572103)	AA	AA	AA	AA	AA
	1196A/G (rs10509681)	TT	TT	TT	TT	TT
	792C/G (rs1058930)	CC	CC	CC	CC	CC
CYP3A5	6986A/G (rs776746)	AG	AA	GG	AG	AG
	27290C/A (rs28365083)	CC	CC	CC	CC	CC

Supplementary Table 9. Genotyping of patient DNA for single nucleotide polymorphisms (SNPs) in genes involved in pharmacokinetics and pharmacodynamics of pazopanib and those related to the oxidative stress and maintenance of redox balance

		Case				
Gene	SNP	NHT1	NHT2	HT1	HT2	HT3
ABCB1	-129T/C (rs3213619)	CT	TT	TT	TT	TT
	1236T/C (rs1128503)	CC	CT	TT	TT	TT
	2677G/T/A (rs2032582)	AA	AT	GT	GG	GT
	3435C/T (rs1045642)	CC	CT	CT	CC	CT
ABCG2	34G/A (rs2231137)	GG	GG	GG	AG	GG
	421C/A (rs2231142)	AC	AC	AC	CC	AC
SLCO1B1	388A/G (rs2306283)	CC	CC	CC	CT	CC
	521T/C (rs4149056)	CT	CT	CT	CT	CT
VEGFA	-2055C/A (rs699947)	CC	AC	CC	CC	CC
	-1498C/T (rs833061)	TT	CT	CT	TT	TT
	936C/T (rs3025039)	CC	CC	CC	CC	CC
VEGFR2	889C/T (rs2305948)	CT	CT	CC	CC	CC
	1416A/T (rs1870377)	AT	AT	TT	AT	TT
HIF1A	1772C/T (rs11549465)	CC	CC	CC	CC	CC
	1790G/A (rs11549467)	GG	GG	GG	GG	GG
GPX1	14G/C (rs8179169)	GG	GG	GG	CG	GG
	599C/T (rs1050450)	CC	CC	CC	CT	CT
	-46C/T (rs1800668)	CC	CC	CC	CT	CT
TXN2	387+30G/T (rs2281082)	GT	GT	GT	GT	TT
CAT	-844G/A (rs769214)	AA	AG	AG	GG	GG
PPARG	34C/G (rs1801282)	CC	CC	CC	CC	CC
